# The ClpA chaperone and the two adaptor proteins modulate the fate of the model substrate tagged with a SsrA-degron of *Leptospira*

**DOI:** 10.1042/BCJ20253143

**Published:** 2025-08-26

**Authors:** Surbhi Kumari, Manish Kumar

**Affiliations:** 1 Department of Biosciences and Bioengineering, Indian Institute of Technology Guwahati, Guwahati, Assam, 781039, India

**Keywords:** adaptor proteins, caseinolytic protease, Clp ATPase, *Leptospira*, SsrA-tagged substrates

## Abstract

Bacterial caseinolytic protease (Clp) chaperone–protease complexes are essential for the degradation of misfolded and aggregated protein substrates. The spirochaete *Leptospira interrogans* possesses a set of Clp adaptor proteins (ClpS1 and ClpS2) and chaperones (ClpX, ClpA and ClpC), which are believed to associate with two distinct isoforms of ClpP (ClpP1 and ClpP2). This study explores the structural and functional properties of LinClpA, LinClpS1 and LinClpS2 derived from *L. interrogans*. LinClpA, a 740-amino acid protein, features an N-terminal domain and two AAA+ ATPase domains (D-I and D-II), containing conserved motifs critical for ATP binding and hydrolysis. LinClpS1 and LinClpS2 exhibit similar structures, yet they possess distinct binding pockets for N-degron substrates. Biochemical assays indicate that the N-domain-deleted variant of LinClpA (LinClpA^ΔN^) exhibits a nucleotide-induced oligomerization tendency similar to LinClpA’s but demonstrates higher ATPase activity. Interaction studies have shown that LinClpA’s ATPase activity is enhanced in the presence of LinClpP isoforms and inhibited by LinClpS isoforms. In contrast, the activity of LinClpA^ΔN^ remained unaffected by LinClpS1 and LinClpS2, highlighting the significance of the N-domain of LinClpA in adaptor protein interactions. Furthermore, the study predicted and evaluated the role of the C-degron tag called small stable RNA A in facilitating protein degradation by the *L. interrogans* ClpAP1P2 machinery.

## Introduction

Targeted proteolysis is vital in regulating cellular processes, maintaining proteome balance and ensuring protein quality control across all cells [1]. In bacteria, these functions are facilitated by energy-dependent ATPase chaperone–protease complexes, which utilize energy from ATP hydrolysis to unfold and transport protein substrates into the proteolytic chamber of proteases [2–4]. Several proteases, including members of the Clp protease family (ClpAP, ClpXP and ClpYQ), as well as FtsH and Lon, are essential for sustaining protein homeostasis [5,6]. The Lon and FtsH proteases comprise a single polypeptide chain encompassing both ATPase and protease domains. In contrast, the Clp proteases consist of distinct polypeptide chains for their ATPase components (ClpA, ClpX or ClpY) and protease components (ClpP and ClpQ) [7–11].

The tetra-decameric complex EcoClpP, a core proteolytic component of *Escherichia coli*, demonstrates a unique capability to interact with two ATPase chaperone partners, namely EcoClpX and EcoClpA [12]. Upon the binding of nucleotides, EcoClpA assembles into an active hexameric chaperone, thereby facilitating the translocation and unfolding of protein substrates through its translocation channel into the catalytic chamber of EcoClpP [13,14]. This process is driven by the energy released during ATP hydrolysis by EcoClpA’s two ATPase domains. The degradation of proteins necessitates a highly specific recognition mechanism to safeguard essential and properly folded proteins within the bacteria [14]. Certain protein substrates are directly identified by the regulatory components ClpA and ClpX associated with the ATP-dependent EcoClpAP and EcoClpXP proteases [14]. In contrast, other substrates are initially recognized by specialized adaptor proteins that subsequently facilitate their delivery to the respective proteases. Proteins designated for degradation typically possess specific motifs known as degrons, which are located at either the carboxy- or amino-terminal ends [15]. The carboxy-terminal tagging of substrate proteins represents the most thoroughly studied C-degrons in *E. coli* [15]. During protein synthesis, premature mRNA, which lacks a stop codon, results in stalled ribosomes. Trans-translation is required to rescue the stalled ribosomes and tag the nascent polypeptide chain with a specific C-degron for its degradation. In *E. coli*, a small stable RNA A (SsrA), also known as transfer-messenger RNA (tmRNA), works with the EcoSmpB protein to rescue stalled ribosomes. This process restores translation, followed by the release of the ribosome and the production of proteins with a C-terminal SsrA tag [15]. The EcoClpAP and EcoClpXP complexes and two distinct adaptor proteins exhibit varying affinities for SsrA-tagged substrates. The adaptor protein EcoSspB enhances the degradation of SsrA-tagged substrates by facilitating their delivery to EcoClpXP [16]. Conversely, the adaptor protein EcoClpS mitigates the binding and subsequent degradation of SsrA-tagged proteins by the EcoClpAP degradation complex [17]. EcoClpS interacts with the N-domain of EcoClpA, thereby steering the specificity of the EcoClpAP complex towards N-degron-tagged substrates and away from those categorized as C-degron substrates [17,18]. In *E. coli*, destabilizing N-terminal residues, referred to as N-degrons, include phenylalanine (F), tyrosine (Y), tryptophan (W) and leucine (L) [18]. The primary destabilizing residues (F/L) are conjugated to a target protein substrate upon recognition of secondary destabilizing residues (Lysine; K or Arginine; R) by a specific F/L transferase [18].

The EcoClpA belongs to ATPase associated with diverse cellular activities (AAA+) superfamily of proteins and its subunit comprises an N-terminal domain (N-domain) and two ATPase domains, D1 and D2. The N-domain of EcoClpA consists entirely of α-helices, exhibiting a two-fold symmetry between helices H1–H4 and H5–H8. The D1 and D2 domains each include a large sub-domain with Walker A and B motifs alongside a smaller domain [19]. Within the EcoClpA hexamer, the D1 and D2 domains are arranged as homomeric rings stacked vertically. The D2 ring of EcoClpA connects to the EcoClpP component, while the D1 domain is linked to the N-domain through a flexible linker [19]. The N-domain can adopt multiple conformations above the D1 ring, functioning as a mobile element [20]. When the substrate-bound EcoClpS interacts with the highly mobile N-domain of EcoClpA, it facilitates the transfer of the substrate to the ATPase domain [21]. EcoClpS is a small protein characterized by an unstructured N-terminal extension (NTE; amino acids 1–25) and a folded core domain (amino acids 26–106) [22]. The monomeric form of EcoClpS contains a single binding site for peptides that include phenylalanine (F), tyrosine (Y), tryptophan (W) or leucine (L) at the N-terminus. The hydrophobic side chains of the N-degron peptide bind to the hydrophobic pockets within the core region of EcoClpS [23]. The interface between the core region of EcoClpS and the N-domain of EcoClpA is primarily characterized by salt bridges and hydrogen bonds [22]. Upon engagement with the N-domain of EcoClpA, the exposed NTE of EcoClpS interacts with the pore loops present within the ATPase domains of EcoClpA, thereby facilitating the delivery of substrates to the protease complex [21].

The Clp family of proteins comprises several ATPase chaperones and its associated serine protease, ClpP. Based on the number of ATPase domains, these chaperones are divided into class I (ClpA and ClpC) with two ATPase domains and class II (ClpX) with one ATPase domain [24]. The proteolytic complex, ClpXP, is present in most bacteria and has been studied in detail in gram-positive and gram-negative bacteria [25–30]. The other chaperone, ClpA, is present in gram-negative bacteria, while ClpC is found in gram-positive bacteria and cyanobacteria [31].

The ClpCP-mediated protein degradation has been studied in various bacteria, including *Bacillus subtilis*, *Streptomyces hawaiiensis*, *Chlamydia trachomatis*, *Mycobacterium tuberculosis* and *L. interrogans* [32–36]. However, a detailed *in vitro* biochemical characterization of ClpA and its association with Clp protease and adaptor proteins has only been performed in *E. coli* [11,17,18,20,21]. Several *in vivo* studies have been reported regarding ClpA chaperone’s functionality in gram-negative bacteria [37–40]. For instance, in *Helicobacter pylori*, HpyClpA plays a role in providing resistance to oxidative and antibiotic stress, while in *Brucella suis*, BsuClpA was reported to be involved in regulating thermal stress [37,38]. Another study on *Caulobacter crescentus* highlights the importance of CcrClpA in controlling chromosome number and content [39]. In *Xanthomonas campestris*, XcaClpA plays a role in pathogenicity and thermal stress tolerance [40]. In this study, ClpA of *Leptospira*, a spirochaete that causes leptospirosis in humans and animals [41] has been explored. The *Leptospira* genome is predicted to have ten genes that encode for Clp family proteins [42]. These genes include two adaptor protein-encoding genes (*clpS1; LIC11356* and *clpS2; LIC11815*), five genes coding for AAA + ATPase (*clpA; LIC11814, clpB; LIC12017, clpC; LIC10339, clpX; LIC11418* and *clpY; LIC11601*), and three genes coding for Clp protease (*clpP1; LIC11417, clpP2; LIC11951,* and *clpQ; LIC11600*) [42]. In a prior study, the *clpB* (*LIC12017*) was reported to be essential for the survival of *Leptospira* under thermal and oxidative stress conditions [42]. Biochemical analysis of LinClpB (LIC12017) has shown that nucleotide induces the formation of LinClpB hexamers with ATPase and disaggregase activity [43]. Additionally, the core catalytic components of the Clp system in *Leptospira* (LinClpP1 and LinClpP2) have been well studied and characterized, revealing that the pure LinClpP1 and LinClpP2 isoforms are catalytically inactive, while the heterocomplex, LinClpP1P2, shows peptidase activity on the di-peptide model substrate [44]. The modulating role of the natural antibiotic acyldepsipeptide (ADEP1) on the LinClpP1P2 protease activity has also been recently studied [45,46]. Furthermore, a comprehensive study on LinClpC, a class-I chaperone, has revealed that it has two ATPase domains separated by a middle domain and undergoes nucleotide-induced oligomerization and the LinClpC oligomers interact with both LinClpP isoforms (LinClpP1 and LinClpP2) with similar affinity [36].

This study examined the Clp chaperone, LinClpA, and its associated adaptor proteins (LinClpS1 and LinClpS2) in *L. interrogans*. Our findings indicate that the N-domain of LinClpA does not play a crucial role in its ATPase activity and interaction with LinClpP isoforms. Furthermore, we show that LinClpA undergoes time-dependent auto-degradation when in a complex with LinClpP1P2, and the signal for this degradation is not located within the N-domain. Our study also provides evidence of the functionality of the C-degron (SsrA tag) of *Leptospira*. The enhanced green fluorescent protein (eGFP), which is usually stable, became a model substrate for LinClpAP1P2 machinery when tagged with the predicted LinSsrA (eGFP-SsrA). Additionally, the inhibitory effect of binding with two different adaptor proteins (LinClpS1 and LinClpS2) on the ATPase activity of LinClpA and protease activity of LinClpAP1P2 machinery has been explored.

## Results

### Structural and sequence analysis of the leptospiral Clp ATPase (LinClpA)

**Figure 1:**
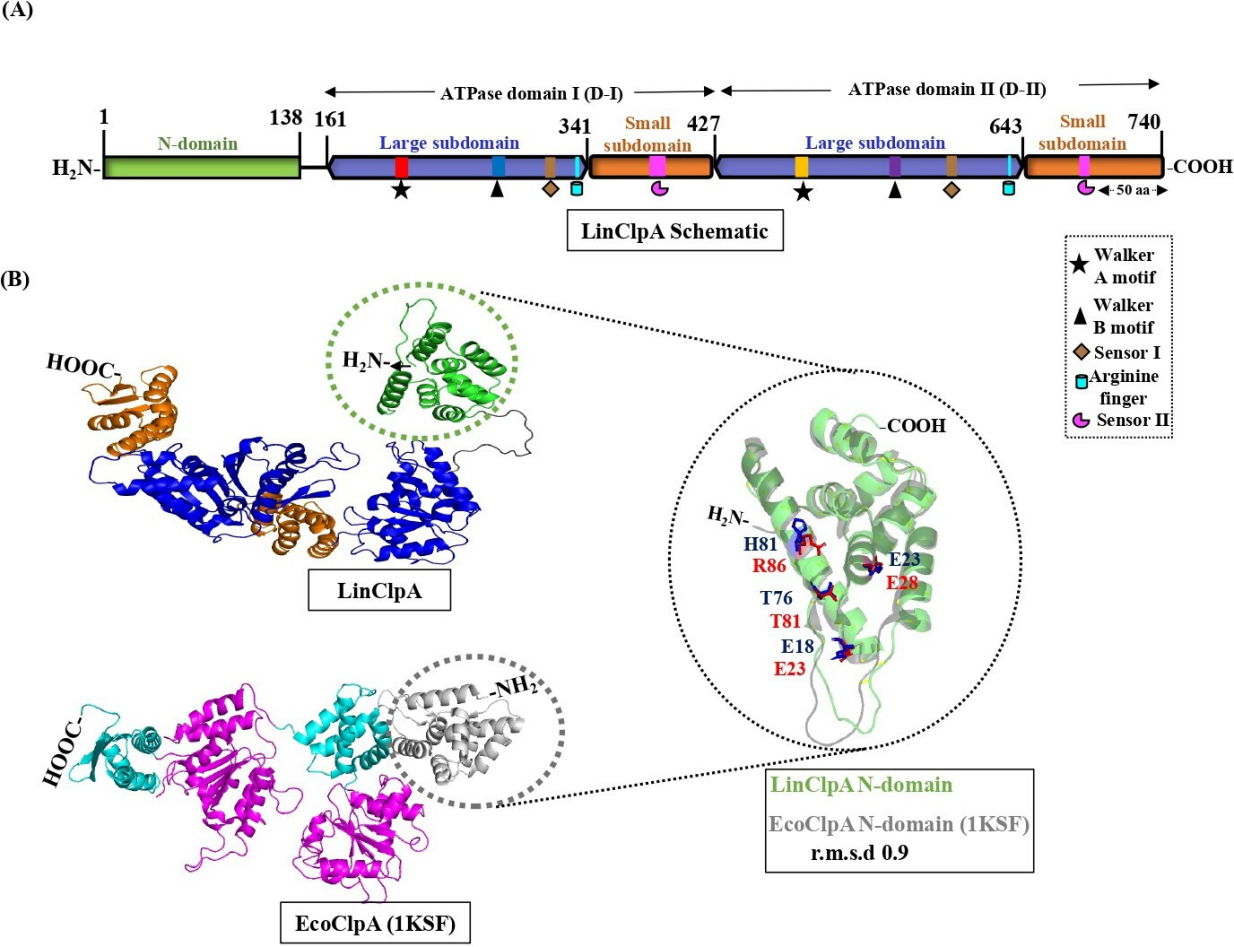
Prediction for LinClpA domain organization and the three-dimensional structure. **(A**) The diagram illustrates the predicted N-domain, ATPase domain I (**D-I**) and ATPase domain II (**D-II**), presented roughly to scale. The large and small subdomains of the ATPase domains are depicted as hexagonal and square shapes, respectively. The essential components of the ATPase domains are colour-coded as follows: Walker A (red and orange), Walker B (blue and violet), Sensor I (brown), Arginine finger (cyan) and Sensor II (magenta). (**B**) The modelled tertiary structure of LinClpA (upper panel) is shown, with the N- and C-terminal ends indicated as NH_2_ and COOH, respectively. The N-domain is illustrated in green, the ATPase large subdomain in blue and the ATPase small subdomain in orange. The tertiary structure of *E. coli* ClpA (EcoClpA; PDB- 1KSF) (lower panel) displays three main components: The N-domain, the ATPase large subdomain and the ATPase small subdomain, represented in grey, magenta and cyan colors, respectively. The N-domains of LinClpA and EcoClpA are highlighted by green and grey circles and zoomed in for structural superimposition. The modelled LinClpA N-domain (green) and EcoClpA N-domain (1KSF; grey) were structurally superimposed with an r.m.s.d. of 0.9. Essential residues for interaction with adaptor proteins are marked as red sticks (EcoClpA N-domain) and blue sticks (LinClpA N-domain).

The full-length LinClpA protein consists of 740 amino acids and includes an N-domain followed by two ATPase domains I and II. Each ATPase domain (I & II) is further subdivided into large and small subdomains (Figure 1A). The N-domain is composed of 138 residues and is connected to a large subdomain by a flexible linker region of 22 residues (from amino acids 139 to 160). The D-I domain spans 267 residues, while the second domain (D-II) comprises 313 residues. A sequence comparison of LinClpA with other bacterial orthologs reveals a high sequence identity with *Pseudomonas aeruginosa* (PaeClpA; 52%), *E. coli* (EcoClpA; 51%) and *X. campestris* (XcaClpA; 50%). In contrast, LinClpA shows lower identity with *H. pylori* (HpyClpA; 38%) and *Borrelia burgdorferi* (BbuClpA; 28%) (Supplementary Figure S1). The pairwise sequence alignment of the N-domain, D-I and D-II of LinClpA and EcoClpA indicates that each ATPase domain shares more than 50% sequence identity, while LinClpA’s N-domain shows 40% identity with EcoClpA’s N-domain. Key elements critical for ATPase function, such as Walker A, Walker B, the Arginine finger, Sensor I and Sensor II, are highly conserved in both the D-I and D-II domains of LinClpA (Figure 1A). The Walker A motif of LinClpA is located in the D-I domain at residues 206–213 (GEAGVGKT) and in the D-II domain at residues 485–492 (GPTGVGKT); this motif is essential for ATP binding and oligomerization. Similarly, the Walker B motif, in the D-I domain of LinClpA at residues 273–280 (VLFIDEIH) and in the D-II domain at residues 551–557 (LLLDEIE), is crucial for ATP hydrolysis. The Sensor I motif and Arginine finger responsible for ATP hydrolysis are located at residues 310–315 (CIGTTT; DI Sensor I) and 591–596 (LVMTTN; DII Sensor I), as well as at Arg331, Arg332 and Arg633, respectively. Additionally, the Sensor II motifs of the D-I and D-II domains of LinClpA, which are involved in conformational changes during ATP binding and hydrolysis, are situated at residues 383–392 (DRKLPDKAID) and 689–694 (FGARPV), respectively. Furthermore, the tertiary structure of the LinClpA monomer was modelled using AlphaFold and showed similarity to the structure of EcoClpA (PDB: 1KSF; Figure 1B). The modelled structure of the LinClpA N-domain consists of eight helices (α1–α8) with approximately 60% helical content, similar to the EcoClpA N-domain [19]. The LinClpA N-domain contains two conserved repeats, each approximately 60 residues long, showing a pseudo-two-fold symmetry. Repeat 1 (α1-α4) can be superimposed onto repeat 2 (α5-α8) of LinClpA with a root mean square deviation (r.m.s.d.) of 1.2 (Supplementary Figure S2A). The large subdomains of D-I and D-II in LinClpA possess a core α/β folding motif characterized by five β-sheets flanked by α-helices on either side, which is analogous to the reported structures of EcoClpA’s D-I and D-II domains (Supplementary Figure S2B, C). [19]. The small subdomain of D-I in LinClpA contains four α-helices, while the small subdomain of D-II has three α-helices and three β-sheets. The N-domain of EcoClpA has multiple binding sites for EcoClpS, which are crucial for EcoClpS docking, followed by substrate recognition and degradation [22]. The structural superimposition of the modelled LinClpA N-domain with EcoClpA N-domain (PDB: 1KSF) indicates that residues facilitating interactions between EcoClpA’s N-domain and EcoClpS are highly conserved in LinClpA’s N-domain (Figure 1B). The predicted contact site with adaptor proteins in LinClpA contains one aliphatic residue (Thr76) and three charged residues (Glu18, Glu23 and His81), which align with EcoClpA N-domain residues (Thr81, Glu23, Glu28 and Arg86) [22].

### Structural and sequence analysis of the leptospiral Clp adaptor proteins (LinClpS1 and LinClpS2)

**Figure 2:**
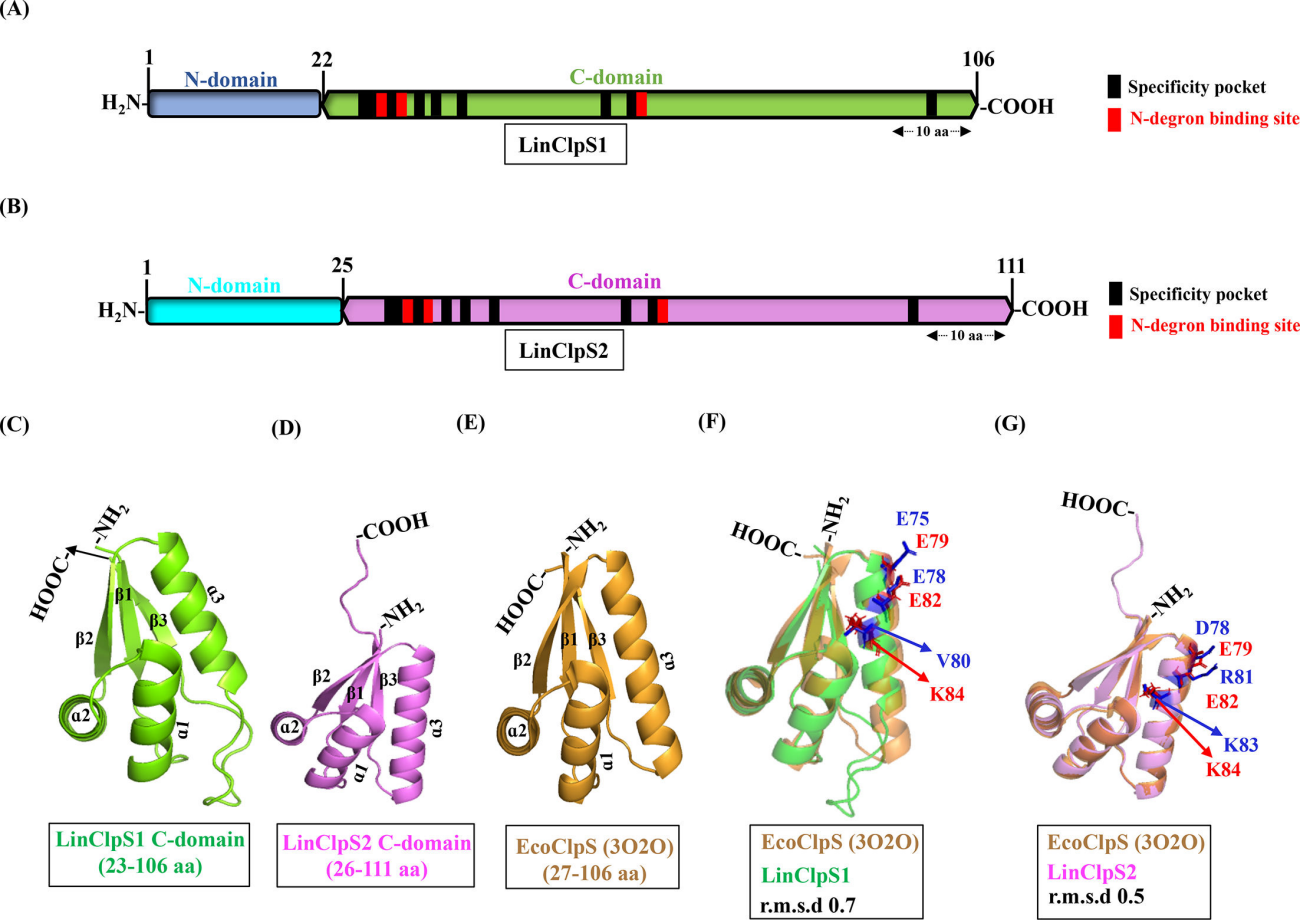
Prediction of the domain organization and three-dimensional structure of LinClpS1 and LinClpS2. The diagram illustrates the predicted domains of (**A**) LinClpS1 and (**B**) LinClpS2 to scale. In the diagram, the N-domains of LinClpS1 and LinClpS2 are represented as square shapes, while the C-domains are depicted as hexagonal shapes. The residues within the C-domain of LinClpS1 and LinClpS2 that are predicted to bind to the LinClpA N-domain are marked with red boxes. Additionally, black boxes in the C-domain indicate the residues likely important for binding with the N-degron peptide (specificity pocket). The modelled tertiary structure of the C-domain of (**C**) LinClpS1 is shown in green, while (**D**) LinClpS2 is represented in magenta. In (**E**), the tertiary structure of *E. coli* ClpS (EcoClpS; 3O2O) is depicted in orange. The structural superimposition in (**F**) demonstrates modelled LinClpS1 (green) and EcoClpS (3O2O; orange) with a r.m.s.d. of 0.7, while (**G**) shows modelled LinClpS2 (magenta) superimposed with EcoClpS (3O2O; orange) with an r.m.s.d. of 0.5. The residues corresponding to the chaperone binding pocket are highlighted as red sticks for EcoClpS and blue sticks for LinClpS1 and LinClpS2

The complete genome sequence of *L. interrogans* shows the existence of two *clpS* genes (*LIC11356; clpS1* and *LIC11815; clpS2*). Interestingly, the genes *clpS2* (333 bp) and *clpA* (2220 bp) in *Leptospira* are situated next to each other on the chromosome with a 14-nucleotide gap, while the *clpS1* (318 bp) is located elsewhere in the genome. LinClpS1 (12 kDa) and LinClpS2 (13 kDa) are quite close in terms of size and structure, where both have an N-domain (22 vs. 25 residues) followed by a C-domain (84 vs. 86 residues) (Figure 2A and B). A comparison of the amino acid sequences of LinClpS1 and LinClpS2 with well-studied ClpS orthologs was carried out using ClustalW and represented as heat map (Supplementary Figure S3A). The length and sequence conservation of the N-domain within various ClpS orthologues vary widely, while the C-domain is moderately conserved. LinClpS2 shares 30–40% sequence identity with EcoClpS, CcrClpS (*C. crescentus* ClpS), AtuClpS1 (*Agrobacterium tumefaciens* ClpS1), AtuClpS2, SelClpS1 (*Synechococcus elongatus* ClpS1). In contrast, LinClpS1 shares a lower sequence identity (15-25%) with EcoClpS, CcrClpS, AtuClpS1, AtuClpS2, SelClpS1, SelClpS2 and MtuClpS (*M. tuberculosis* ClpS). The co-crystal structure of CcrClpS with a decapeptide having Tyr residue at the N-terminal demonstrates that the α-amino group of the peptide interacts with specific residues (Asn47, Asp49 and His79) of CcrClpS [47]. The residues corresponding to the peptide interaction site in CcrClpS are highlighted for LinClpS1 and LinClpS2 (Figure 2A and B). The LinClpS1 protein retains conserved Asp30 and Asn32 residues, while the conserved His residue has been evolutionarily replaced with Asp62 within the binding pocket. The binding pocket of LinClpS2 maintained the conserved residues (Asn33, Asp35 and His65) alike its orthologues. In the CcrClpS-peptide co-crystal, the tyrosine ring of the peptide fits into a deep hydrophobic pocket on the surface of CcrClpS. This hydrophobic pocket for CcrClpS, known as the specificity pocket, is formed by a list of residues (Ile45, Leu46, Asn47, Asp48, Thr51, Met53, Val56, Met75, Val78, His79 and Leu112) [47]. Accordingly, the residues corresponding to the specificity pocket of LinClpS1 (Leu28, Trp29, Asp31, His34, Tyr36, Val39, Ala58, Val61, Met100) and LinClpS2 (Ile31, Leu32, Asp34, Thr37, Met39, Val42, Met61, Ala64, Leu98) were assessed and highlighted (Figure 2A and B). In EcoClpS of *E. coli*, the residue Met40 acts as a specific gatekeeper, accommodating the hydrophobic side chains of a few residues (Phe, Trp, Tyr and Leu) while excluding β-branched amino acid-containing peptides [47]. The residue Met40 of the specificity pocket recorded in EcoClpS is also conserved in LinClpS2; however, LinClpS1 has a non-conserved residue Tyr36. The natural replacement of Met with Tyr residue in the LinClpS1 peptide binding pocket might indicate a different substrate specificity compared with EcoClpS, CcrClpS and LinClpS2.

The three-dimensional structure of LinClpS1 and LinClpS2 was modelled using AlphaFold and compared with the available crystal structure of EcoClpS (PDB: 3O2O) (Figure 2C–E). EcoClpS has a disordered N-domain, referred to as the NTE, and a folded core domain (26–106 residues) [22]. The disordered region in the N-domain of LinClpS1 and LinClpS2 was assessed using the PrDOS online server and compared with EcoClpS [48]. The server predicts the disordered regions of a protein from its amino acid sequence and provides a disorder probability for each residue. The average disorder probability of the N-domain of LinClpS1, LinClpS2 and EcoClpS was estimated to be 0.6–0.7 (Supplementary Figure S3B). This suggests that LinClpS1 and LinClpS2 also possess an unstructured NTE region similar to EcoClpS [22].

The modelled structures of LinClpS1 (23–106 residues) and LinClpS2 (26–111 residues) are composed of three α-helices (α1–α3) linked to three β-strands (β1–β3) in a βααβαβ arrangement (Figure 2C and D). In EcoClpS, the helix α3 contains highly conserved residues (Glu82 and Lys84), which form salt bridges with the EcoClpA residues, Arg86 and Glu23, respectively [22]. Additionally, the Glu79 of EcoClpS forms hydrogen bonds with Glu28 and Thr81 of EcoClpA [22]. The modelled structures of LinClpS1 and LinClpS2 were superimposed with EcoClpS (PDB: 3O2O), with r.m.s.d. values of 0.7 and 0.5, respectively (Figure 2F and G). The structure comparison revealed that in LinClpS1, the conserved charged residue Lys84 present in EcoClpS has been evolutionarily replaced with the aliphatic Val80, while LinClpS2 retains the conserved Lys83 residue. These variations within the chaperone binding pocket of LinClpS1 and LinClpS2 may indicate their different affinities for the N-domain of LinClpA. To comprehensively study Clp ATPase and adaptor proteins of *Leptospira*, the genes of interest (*clpA*, *clpS1* and *clpS2*) were cloned, and recombinant proteins were over-expressed and purified from *E. coli* BL21 cells. A partial *clpA* gene (*clpA^∆N^*) encoding LinClpA with a deleted N-domain (138 residues) was also cloned, over-expressed and purified. The quality of the recombinant proteins (LinClpA, LinClpA^∆N^, LinClpS1 and LinClpS2) purified using Ni-NTA affinity chromatography was analysed on a polyacrylamide gel and stained with Coomassie Blue (Supplementary Figure S3C). Polyclonal antibodies were generated against recombinant LinClpA protein in BALB/c mice, and the resulting serum was evaluated for antibody titre determination. Following this, immunoblot analysis was conducted using whole-cell lysates of *L. interrogans* along with the corresponding recombinant protein. The LinClpA antibodies successfully detected the native LinClpA protein, which has an approximate molecular weight of 82 kDa, expressed in *L. interrogans* serovar Copenhageni. Furthermore, the immunoblot results demonstrated the specificity of the LinClpA antibodies, as no cross-reactivity was observed with other related proteins, such as the LinClpP isoforms or the LinClpC chaperone present in the whole-cell lysate of serovar Copenhageni (Supplementary Figure S3D).

### Analysis of nucleotide-induced oligomerization of LinClpA

**Figure 3:**
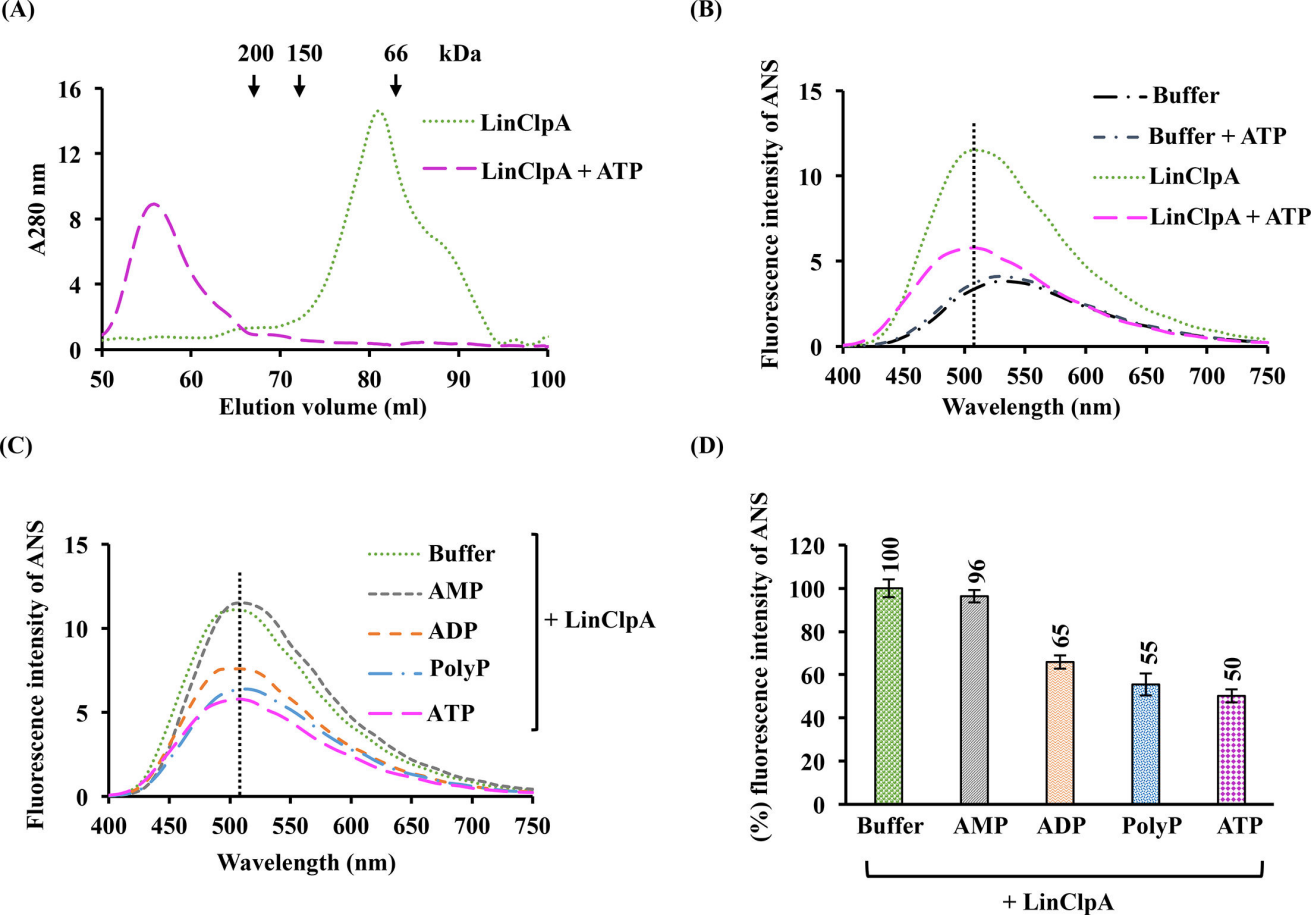
Oligomerization property of LinClpA. **(A**) Size exclusion chromatography analysis of LinClpA in the absence (-) and presence (+) of ATP. An ANS binding assay was conducted to study the oligomerization of LinClpA. This assay measured the emission spectra of a hydrophobic fluorophore, ANS, in the 400–750 nm range, using an excitation wavelength of 350 nm. The fluorescence intensity of ANS was recorded when bound to LinClpA (**B**), both in the absence (-) and presence (+) of ATP (**C**), alongside other molecules such as AMP, ADP and PolyP. (**D**) The percentage change in fluorescence intensity of ANS was calculated when LinClpA was bound to AMP, ADP, PolyP and ATP against a sample of pure LinClpA, which served as the baseline (considered as 100%). Each ANS binding assay experiment was performed three times, and one representative spectrum was displayed.

The AAA+ proteins typically assemble into higher oligomeric structures upon binding to nucleotides [49]. The effect of nucleotide (ATP; 2 mM) on the oligomeric state of purified recombinant LinClpA was analysed using size-exclusion chromatography (Figure 3A). In the absence of ATP, LinClpA predominantly eluted in its monomeric form, approximately 82 kDa. However, when incubated with ATP, the elution profile shifted towards a higher molecular weight species, around 492 kDa, indicating that ATP induces the oligomerization of LinClpA. Furthermore, previous research has explored the oligomerization propensity of a leptospiral ATPase chaperone, LinClpC, using a hydrophobic fluorophore 8-anilino-1-naphthalenesulphonic acid (ANS) dye-binding assay [36]. To examine the nucleotide-induced oligomerization of LinClpA, a binding assay utilizing the ANS dye was also conducted (Figure 3B). ANS is inclined to bind to the exposed hydrophobic regions of proteins, resulting in increased fluorescence than the unbound form. Oligomerization of proteins leads to the masking of hydrophobic regions, causing reduced binding with ANS [50]. Pure LinClpA or LinClpA pre-incubated with ATP was mixed with ANS in assay buffer, and fluorescence spectra were examined at a fixed excitation wavelength of 350 nm and emission wavelength ranging from 400 to 750 nm (Figure 3B). Pre-incubation of LinClpA with ATP resulted in the reorganization of its subunits into higher molecular weight complexes, leading to reduced binding with ANS and a consequent decrease in fluorescence intensity, indicating higher-order oligomerization of LinClpA in the presence of ATP. To determine whether the oligomerization of LinClpA is specifically induced by ATP or whether it can also occur in the presence of other nucleotides, ANS binding assays were conducted using either CTP, GTP or UTP. Pre-incubation of LinClpA with ATP, GTP, CTP or UTP (at a concentration of 2 mM) resulted in a decrease in ANS fluorescence intensity, indicating oligomer formation compared with the unbound LinClpA. These findings suggest that LinClpA does not have strict nucleotide specificity and can interact with various nucleotides, including GTP, CTP and UTP, in addition to ATP (Supplementary Figure S4A). Previous reports have indicated that EcoClpA forms hexamers upon binding with nucleotide triphosphates (ATP or ATPγS), while the addition of nucleotide diphosphate (ADP) promotes its dissociation into dimers [49,51]. Another study on EcoClpA oligomerization suggests that the oligomerization of EcoClpA is induced by nucleotide triphosphate analogues (ATPγS, AMP-PNP and AMP-PCP) as well as nucleotide diphosphates (ADP·BeF and ADP) [52]. These significant differences in the oligomerization behaviour of EcoClpA prompted an investigation into the oligomerization tendency of LinClpA in the presence of various nucleotide analogues. The fluorescence spectra of LinClpA incubated with ATP, ADP, AMP or polyphosphates (PolyP) were analysed and compared with pure LinClpA (Figure 3C). The spectral analysis revealed that incubation of LinClpA with ATP, ADP or PolyP promoted its oligomerization into higher molecular weight complexes and resulted in a reduction of ANS fluorescence. Conversely, pre-incubation of LinClpA with AMP resulted in no spectral change compared to pure LinClpA. Furthermore, the percentage change in the fluorescence intensity of ANS for each reaction was determined using the fluorescence intensity of ANS bound to pure LinClpA as the reference (Figure 3D). The findings showed that pre-incubation of LinClpA with ADP, PolyP or ATP led to reductions in ANS fluorescence intensity from 100% to 65%, 55% and 50%, respectively. Therefore, it can be concluded that the presence of two or more phosphate moieties (ATP, ADP or PolyP) promotes the oligomerization of LinClpA, while AMP with only one phosphate does not have the same effect.

### LinClpA intrinsic ATPase activity

**Figure 4:**
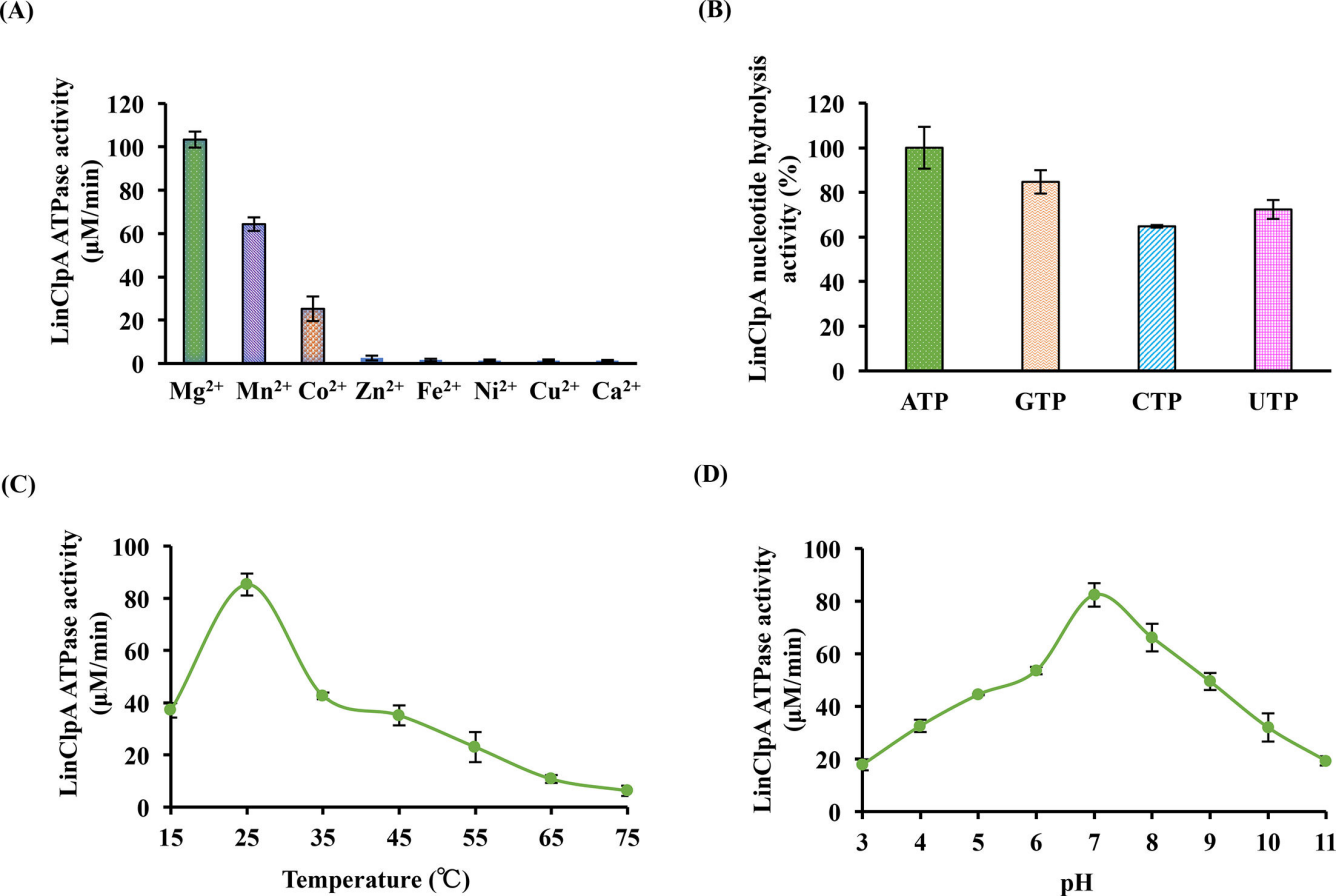
Nucleotide hydrolysis property of LinClpA under diverse parameters. **(A**) LinClpA ATPase activity was measured in the presence of various divalent metal cations (Mg^2+^, Mn^2+^, Co^2+^, Zn^2+^, Fe^2+^, Ni^2+^, Cu^2+^ and Ca^2+^). (**B**) The nucleotide hydrolysis activity of LinClpA on various nucleotides (GTP, CTP, UTP) was compared with the rate of hydrolysis of ATP (considering 100%). (**C**) LinClpA ATPase activity in the temperature range 25–75°C (**D**) LinClpA ATPase activity over a broad pH range 3–11. The data represent the mean ± standard error mean (SEM) from the three independent experiments.

A malachite green assay was utilized to quantify the intrinsic ATP hydrolysis activity of LinClpA, which detects the liberated phosphate subsequent to ATP hydrolysis into ADP. First, the influence of different divalent metal cations on LinClpA’s (1 μM) ATP hydrolysis activity was investigated for 1 hour at 37°C by introducing various metal ions (8 mM) to the buffer (50 mM Tris-Cl, 50 mM KCl, 1 mM DTT; pH 7.8) (Figure 4A). The highest observed LinClpA ATPase activity occurred in the presence of Mg^2+^ (8 mM). Under similar conditions, when equimolar (8 mM) amounts of either Mn^2+^ or Co^2+^ were utilized, the ATPase activity of LinClpA was only 64% and 25%, respectively, compared with that of Mg^2+^ (100%). Other tested cations (Zn^2+^, Fe^2+^, Ni^2+^, Cu^2+^ and Ca^2+^) did not support LinClpA ATPase activity (Figure 4A).

Subsequently, the investigation into the release of phosphate during the hydrolysis of various nucleotides, including GTP, CTP and UTP, was aimed at evaluating LinClpA’s ability to hydrolyze these nucleotides compared with ATP. Although LinClpA primarily utilizes ATP as its substrate, it was determined that it could also hydrolyze other triphosphates: GTP, CTP and UTP (Figure 4B). Specifically, LinClpA demonstrated phosphate hydrolysis rates of 84% for GTP, 64% for CTP and 72% for UTP, with ATP serving as the standard at 100%. Furthermore, the ATPase activity of LinClpA was examined to understand how environmental factors, such as pH and temperature, influence its functionality. Optimal ATPase activity was observed within a temperature range of 20–30°C and at a pH of 7 (Figure 4C and D).

A comparison of nucleotide-induced oligomerization between LinClpA and LinClpA^∆N^ revealed no significant differences, suggesting that the N-domain is not critical for the protein’s oligomerization (Supplementary Figure S4B). Additionally, both LinClpA and LinClpA^∆N^ demonstrated dose-dependent ATPase activity, reaching saturation at specific ATP concentrations (Supplementary Figure S4C). The maximum activity for LinClpA was recorded at 94.6 ± 4 μM/min, while for LinClpA^∆N^, it was 102 ± 4.5 μM/min, indicating a 15% increase in V_max_ for LinClpA^∆N^.

### Differential association of LinClpA and LinClpA^∆N^ with Clp protease and adaptor proteins

**Figure 5:**
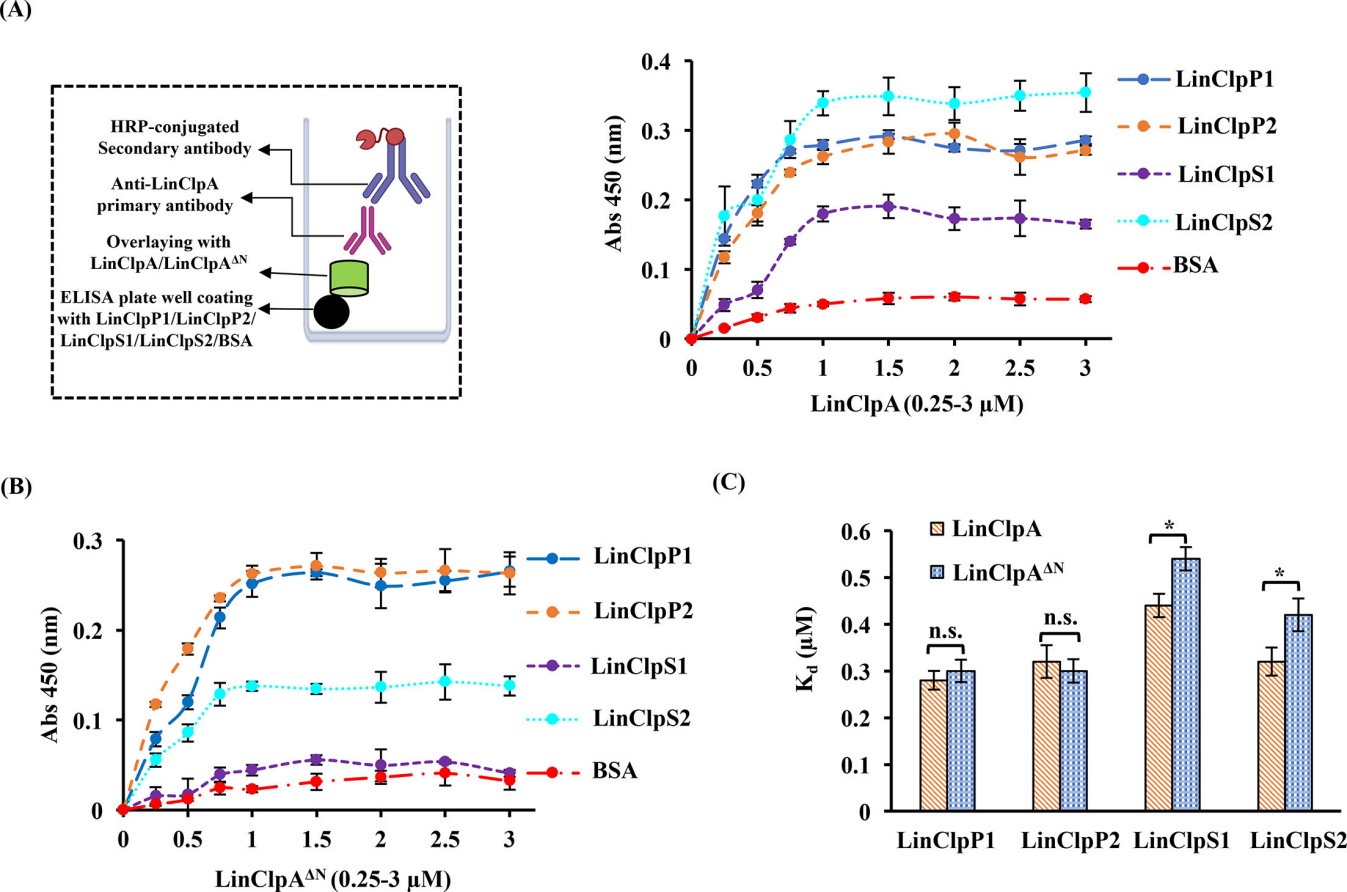
Association of LinClpA or its variant LinClpA^∆N^ with LinClp protease and adaptor proteins. The interaction study used enzyme-linked immunosorbent assay (ELISA) under *in vitro* conditions. The interaction between (**A**) LinClpA and LinClpP1/LinClpP2 or LinClpS1/LinClpS2 or BSA, (**B**) LinClpA^∆N^ and LinClpP/LinClpP2 or LinClpS1/LinClpS2 or BSA. (**C**) The dissociation constant (*Kd*) of the complexes (LinClpAP1, LinClpAP2, LinClpAS1, LinClpAS2 and LinClpA^∆N^P1, LinClpA^∆N^P2, LinClpA^∆N^S1, LinClpA^∆N^S2) was determined via Hill plot analysis and plotted. The data represent the mean ± standard error of the mean (SEM) from three independent experiments.

A study was conducted to assess the capacity of LinClpA to form complexes with the LinClpP isoforms (LinClpP1 and LinClpP2), and this was compared with the association abilities of LinClpA with the N-domain-deleted variant (LinClpA^ΔN^). To assess the specificity of the LinClpA antibodies, we performed a direct enzyme-linked immunosorbent assay (ELISA). In this procedure, microtiter wells were coated with various recombinant proteins from the LinClp system (1 µM; ClpP1, ClpP2, ClpS1, ClpS2, ClpA and ClpA^ΔN^), followed by detection using the LinClpA antibodies (Supplementary Figure S5A). The results showed no significant binding of the antibodies to either ClpP isoforms or ClpS proteins, indicating that the anti-LinClpA antibodies do not exhibit non-specific interactions with these related proteins. Next, an ELISA experiment using antibodies against LinClpA (anti-LinClpA) was employed to monitor the interactions of ATP-bound LinClpA or LinClpA^ΔN^ with the LinClpP isoforms. The results demonstrated a linear increase in absorbance at 450 nm for the complexes (LinClpAP1, LinClpAP2, LinClpA^ΔN^P1 and LinClpA^ΔN^P2) as the concentrations of LinClpA and LinClpA^ΔN^ increased, as shown in Figure 5A and B. The dissociation constant (*Kd*) for these complexes was determined through a Hill plot analysis, and the obtained *Kd* values for the complexes are displayed in Figure 5C. Our immunoassay results indicate that LinClpA and LinClpA^ΔN^ exhibit similar affinities for the pure LinClpP1 and LinClpP2 isoforms, as reflected by a *Kd* value of 0.28 ± 0.02 μM. We also investigated the specificity of the LinClpA–LinClpP (1 μM) interactions in both the presence and absence of various nucleotides (2 mM: ATP, CTP, GTP and UTP) (Supplementary Figure S5B). Our findings indicate that nucleotide-bound LinClpA interacts with LinClpP isoforms at a level comparable to that of nucleotide-free LinClpA. This implies that the presence of nucleotides has a minimal effect on the formation of LinClpAP1 and LinClpAP2 complexes.

Furthermore, an immunoassay was performed to explore the association between LinClpS adaptor proteins (LinClpS1 and LinClpS2) and LinClpA or LinClpA^∆N^ (Figure 5A–B). The results revealed that LinClpA exhibits differential interactions with LinClpS1 and LinClpS2. Notably, a lower dissociation constant was observed for the LinClpAS2 complex (0.32 ± 0.03 μM) relative to the LinClpAS1 complex (0.44 ± 0.025 μM), indicating that the LinClpAS2 complex is more stable than its LinClpAS1 counterpart. Furthermore, a significant change in the dissociation constants for the complexes LinClpA^∆N^S1 and LinClpA^∆N^S2 was noted when compared with LinClpAS1 and LinClpAS2, respectively (Figure 5C). Interestingly, even upon deletion of the N-domain of LinClpA, LinClpS2 continued to show moderate interactions with the LinClpA^∆N^ variant, whereas the interaction with LinClpS1 was completely abolished (Figure 5B). This observation suggests that LinClpS2 may interact with additional sites within LinClpA beyond the N-domain and further investigations are required to validate this hypothesis and to identify potential alternative binding sites that may mediate the strong interaction between LinClpA and LinClpS2. These findings suggest that the presence of the N-domain in LinClpA is essential for stable complex formation with LinClpS adaptor proteins, while it does not affect the formation of complexes with LinClpP isoforms.

**Figure 6:**
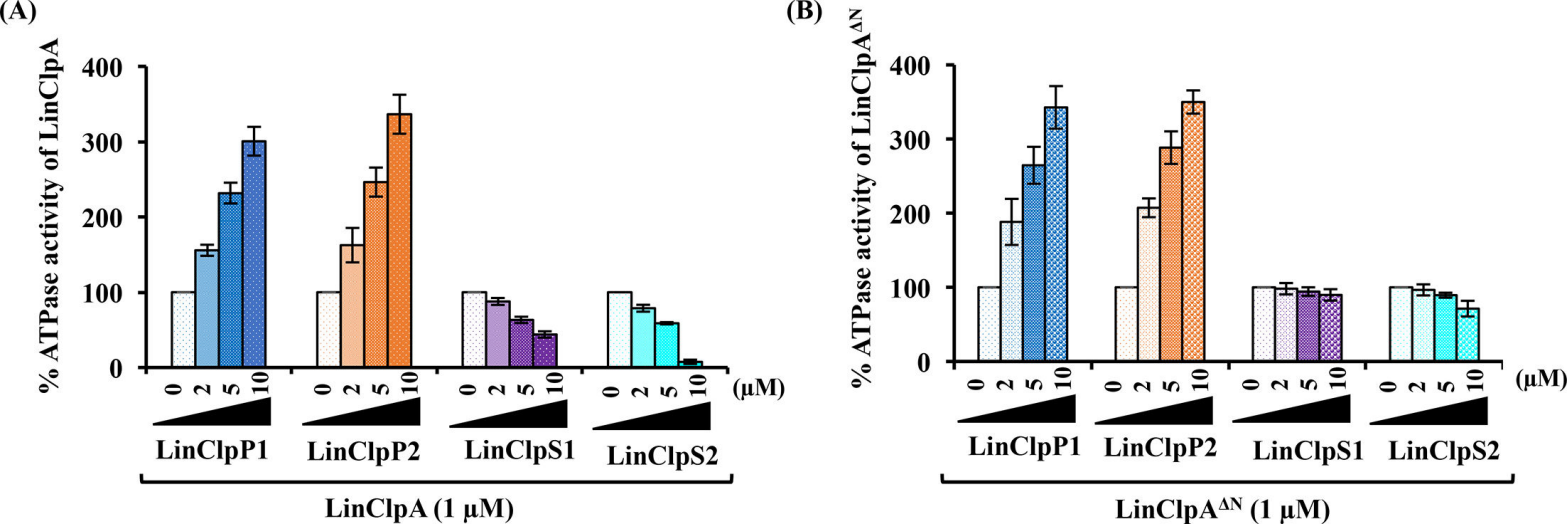
Influence of LinClpP and LinClpS on the ATPase activity of LinClpA and its variant. The ATPase activity of (**A**) LinClpA and (**B**) LinClpA^∆N^ was measured in the presence of increasing concentrations of either LinClpP1, LinClpP2, LinClpS1 or LinClpS2. The percentage change in ATPase activity of LinClpA in the presence of an activator (LinClpP1 and LinClpP2) or inhibitor (LinClpS1 and LinClpS2) was calculated considering the activity of pure LinClpA to be 100%. The data represent the mean ± standard error mean (SEM) from the three independent experiments.

Furthermore, we confirmed the impaired interaction between LinClpA^∆N^ and LinClpS adaptor proteins through ATPase activity analysis. We assessed the rate of ATP hydrolysis by LinClpA and LinClpA^∆N^ in the presence of various LinClpP isoforms or LinClpS adaptor proteins (Figure 6A–6B). Our findings indicate that as the concentration of pure LinClpP isoforms increased, both LinClpA and LinClpA^∆N^ exhibited a linear rise in ATPase activity. In contrast, LinClpA’s ATPase activity was inhibited in a dose-dependent manner by either LinClpS1 or LinClpS2. Adding either LinClpS1 or LinClpS2 (10 μM) resulted in a 57% and 90% reduction in the LinClpA ATPase activity, respectively. Thus, the LinClpAS2 demonstrated a more stable complex than LinClpAS1 (Figure 6A). Nevertheless, the presence of LinClpS1 or LinClpS2 did not affect the ATPase activity of LinClpA^∆N^, which supports the need for the adaptor protein to bind to the N-domain of the ATPase chaperone (Figure 6B).

### Auto-degradation of ClpA is retained in the absence of its N-domain

**Figure 7:**
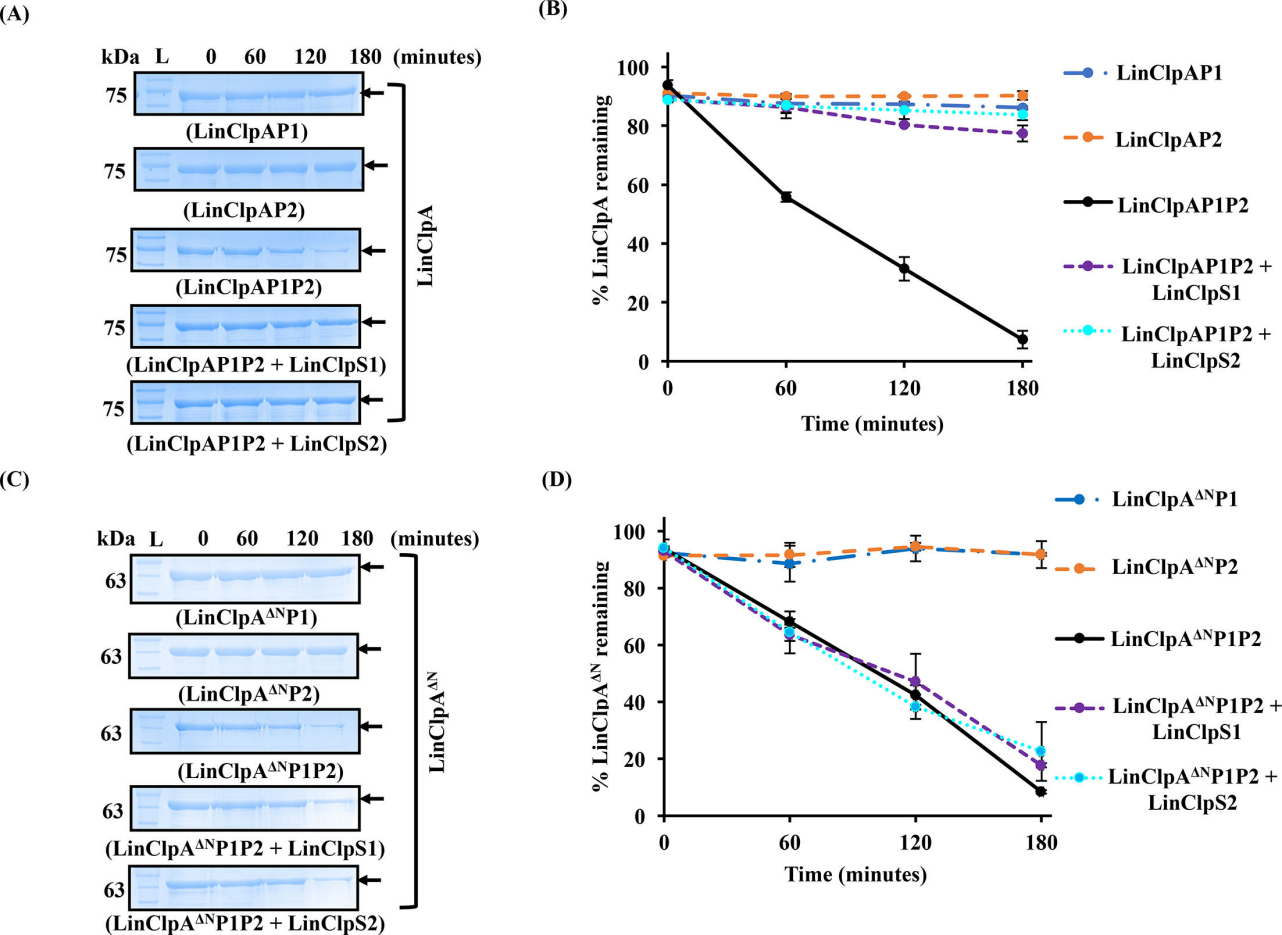
LinClpS inhibits LinClpP1P2-mediated auto-degradation exclusively to LinClpA, not its variant. **(A**) The LinClpA auto-degradation was checked using different self-assembled complexes (LinClpAP1, LinClpAP2 or LinClpAP1P2) in the presence of ATP for 180 minutes. The reaction was supplemented with LinClpS1 or LinClpS2, wherever required. (**B**) The remnant LinClpA was estimated by densitometry analysis using ImageLab software of the Coomassie-stained LinClpA bands in the SDS-PAGE gel. The percentage of LinClpA remaining at different time points of the reaction was plotted. (**C**) The LinClpA^∆N^ auto-degradation was checked using different self-assembled complexes (LinClpA^∆N^P1, LinClpA^∆N^P2 or LinClpA^∆N^P1P2) in the presence of ATP for 180 minutes. The reaction was supplemented with LinClpS1 or LinClpS2, wherever required. (**D**) The remnant LinClpA^∆N^ in the reaction was determined by densitometry analysis using ImageLab software of the Coomassie-stained LinClpA^∆N^ bands.

In *E. coli*, the chaperone EcoClpA has been recognized as a substrate for degradation by EcoClpAP under *in vivo* and *in vitro* conditions [53,54]. Once the natural substrate is depleted, EcoClpA becomes the target and undergoes *in vitro* degradation in a time- and ATP-dependent manner, by EcoClpAP [53]. Therefore, there was a strong interest in investigating whether LinClpA exhibits auto-degradation in the absence of substrates when interacting with the LinClpP1P2 complex. The leptospiral Clp system contains two ClpP isoforms, LinClpP1 and LinClpP2, which necessitated the initial examination of LinClpA’s auto-degradation using these pure LinClpP isoforms in their respective complexes (Figure 7A). As expected, the association of LinClpA with the pure LinClpP isoforms did not confer any activity to the complex. Subsequently, we assessed the time-dependent auto-degradation of LinClpA by the LinClpAP1P2 machinery. This reaction involved mixing the self-assembled LinClpP1P2 heterocomplex with ATP-bound LinClpA and incubating it for 180 minutes at 37°C. At intervals of 0, 60, 120 and 180 minutes, 25 μl of the reaction mixture was resolved on a polyacrylamide gel and stained with Coomassie blue. The LinClpAP1P2 complex demonstrated a time-dependent auto-degradation of LinClpA on the polyacrylamide gel. Densitometry analysis of the LinClpA protein band, depicted in Figure 7A, was conducted at various time points and represented as a percentage of LinClpA remaining over time (Figure 7B). A 90% reduction in the band intensity of LinClpA was observed after 180 minutes. These findings prompted further investigation into the effects of adaptor proteins (LinClpS1 and LinClpS2) on LinClpAP1P2-mediated auto-degradation of LinClpA. Consistent with previous studies on EcoClpA, both LinClpS1 and LinClpS2 inhibited the auto-degradation of LinClpA mediated by the LinClpAP1P2 complex (Figure 7A). It was noted that the adaptor proteins remained stable throughout the experiment, suggesting that they do not directly compete with the ATPase chaperone to inhibit LinClpA’s auto-degradation. Interestingly, LinClpA^∆N^ also displayed time-dependent auto-degradation by the LinClpA^∆N^P1P2 complex (Figure 7C–D). This indicates that the N-domain of the ATPase chaperone has an insignificant role in modulating the protease activity of the core ClpP protease. However, the auto-degradation of LinClpA^∆N^ was not inhibited in the presence of LinClpS by the LinClpA^∆N^P1P2 complex. Therefore, it can be concluded that the binding of LinClpS to the N-domain of LinClpA is necessary to prevent auto-degradation.

### *ssrA* gene of *Leptospira* encoding C-degron mediates rapid degradation of eGFP-SsrA substrate

The substrate specificity of the leptospiral Clp system and its associated mechanisms remain unexplored. The ClpAP system of *E. coli*, which has been extensively studied, is known to be involved in degrading substrates with specific degradation tags located at the amino-terminal (N-degron tag) or carboxy-terminal (SsrA tag) [55,56]. Proteins starting with primary destabilizing residues (N-degrons; Phe, Leu, Trp or Tyr) are recognized by the binding pocket on the core region of the EcoClpS adaptor. This adaptor delivers the proteins to EcoClpAP for degradation [55]. In *E. coli*, proteins starting with N-terminal Arg or Lys residues are recognized by leucyl/phenylalanyl-tRNA-protein transferase (LFTR transferase), which conjugates the protein with N-terminal Leu or Phe residue for recognition by EcoClpS [57]. Another primary destabilizing residue transferase, aspartate/glutamate leucyltransferase (Bpt), has been reported in *Vibrio vulnificus*. This Bpt transferase conjugates a Leu residue to proteins beginning with Asp or Glu [58]. A preliminary *Leptospira* genome analysis suggested genes encoding for both LFTR (*LIC10096*; 660 bp) and Bpt (*LIC11930*; 774 bp) transferases are present. The functionality of these genes encoding for transferases for protein homeostasis in spirochetes is warranted to be explored in future studies.

**Figure 8:**
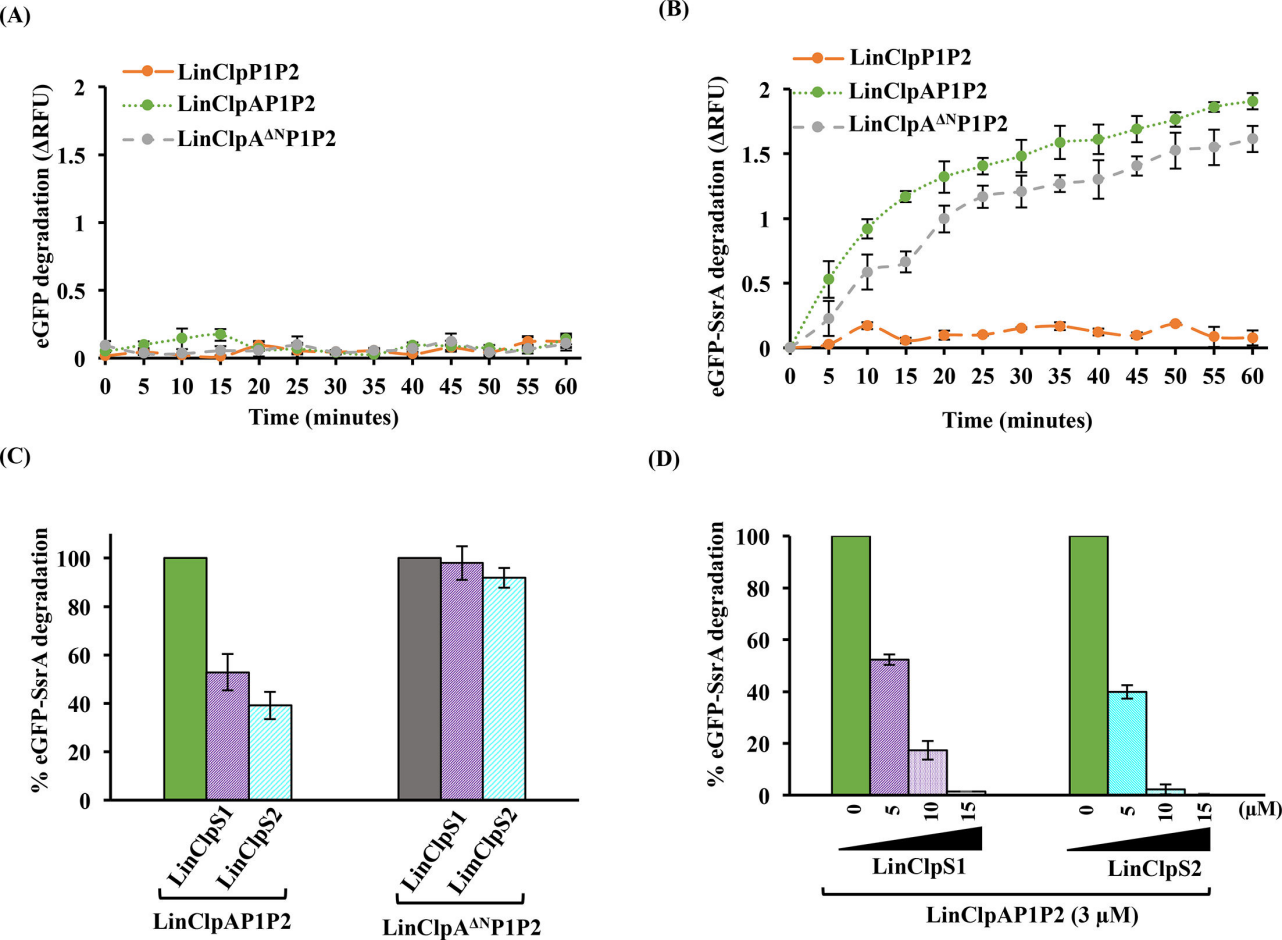
Proteolysis of the model substrate (eGFP) tagged with predicted leptospiral SsrA tag. The degradation of (**A**) eGFP and (**B**) eGFP-SsrA was monitored by measuring fluorescence intensity at excitation and emission wavelengths of 485 nm and 525 nm, respectively, in the presence of various self-assembled complexes (LinClpP1P2, LinClpAP1P2 and LinClpA^∆N^P1P2). The eGFP or eGFP-SsrA fluorescence intensity change (∆RFU) was calculated using RFU_INITIAL_ – RFU_FINAL_ and plotted with time. (**C**) The LinClpAP1P2 or LinClpA^∆N^P1P2-mediated degradation of eGFP-SsrA was measured after 60 minutes in the presence of either LinClpS1 or LinClpS2 (5 µM). (**D**) The LinClpAP1P2 proteolytic activity on eGFP-SsrA substrate in the presence of LinClpS proteins at increasing concentrations (5, 10, 15 µM) was measured after 60 minutes. The change in the degradation rate of eGFP-SsrA with LinClpS1 or LinClpS2 addition was calculated considering LinClpAP1P2 or LinClpA^∆N^P1P2 machinery activity as 100%.

The SsrA tag is utilized to rescue stalled ribosomes during protein synthesis in prokaryotes, particularly in the presence of premature mRNA [59]. The stalled ribosome is recognized by a hybrid RNA called tmRNA encoded by the *ssrA* gene in association with the SmpB protein [59]. This recognition facilitates the completion of translation and subsequent ribosome release [59]. The resulting polypeptide, marked with a C-terminal SsrA tag, is targeted for degradation by ClpAP and ClpXP in *E. coli* or ClpXP in *B. subtilis* [15,60]. The genome of *L. interrogans* serovar Copenhageni is predicted to contain the *ssrA* gene (348 bp) and its associated *smpB* gene (*LIC12418*; 480 bp). While the sequences of C-terminal proteolytic tags encoded by the *ssrA* gene from various bacterial species have been listed in the tmRNA database, their potential in targeting polypeptides for proteolysis remains to be evaluated [54]. The functionality of the SsrA tag has been extensively studied and established in selected bacteria such as *E. coli*, *B. subtilis*, *S. aureus*, S. *pneumoniae* and *M. tuberculosis* [56,60–63]. This study compared the SsrA-tag sequences of several bacterial species with well-characterized ClpP systems (Table 1). The length of the SsrA tag varies significantly among organisms, ranging from 9 residues (*L. interrogans*, *M. tuberculosis* and *C. difficile*) to 22 residues (*C. trachomatis*) [64]. WebLogo representation was used to compare the sequence of the last 9 residues of the SsrA tag from the listed bacteria (Supplementary Figure S6A) [65]. Notably, the last two Ala residues are conserved across all sequences and are crucial for docking onto the EcoClpX ATPase chaperone [66]. Additionally, the critical residues for recognition by EcoClpA in the 10-amino acid-long SsrA tag of *E. coli* were reported to be Ala-8, Leu-9 and Ala-10 [66]. The last four residues of the leptospiral SsrA sequence (ANNELALAA), which resemble those in *E. coli* (ANDENYALAA), may be involved in the association of SsrA-tagged substrates with LinClpA. To study the role of the leptospiral ClpAP1P2 machinery in degrading proteins tagged with SsrA, we created an eGFP-SsrA model substrate. This was achieved by inserting the DNA sequence encoding the predicted amino acid sequence (ANNELALAA) of SsrA from *Leptospira* into the pET21d-eGFP plasmid. This modification allows for the expression of the eGFP protein, which is tagged with SsrA at its C-terminus. Subsequently, the recombinant eGFP and eGFP-SsrA with N-terminal 6 × His tags were purified by Ni-NTA affinity chromatography. The *in vitro* degradation of the eGFP and eGFP-SsrA substrates was assessed by LinClpP1P2 heterocomplex alone or in association with ATP-bound LinClpA or LinClpA^∆N^ (LinClpAP1P2 and LinClpA^∆N^P1P2 machinery) (Figure 8A and B). The incubation of the eGFP-SsrA substrate with the LinClpAP1P2 machinery resulted in a noticeable decrease in fluorescence over time, while the eGFP retained stable fluorescence intensity. In *E. coli*, *in vitro* studies have demonstrated that the auto-degradation of EcoClpA by the EcoClpAP complex is inhibited in the presence of SsrA-tagged model substrates [67]. In a similar investigation, we conducted a degradation assay utilizing the LinClpAP1P2 complex with eGFP-SsrA as the substrate and analysed the reaction products via SDS-PAGE (Supplementary Figure S6B). We observed a time-dependent decrease in the intensity of the eGFP-SsrA band, which confirmed the proteolytic activity of the LinClpAP1P2 machinery. Notably, the auto-degradation of LinClpA was suppressed in the presence of the eGFP-SsrA substrate, indicating that the substrate offers protection against self-degradation. Furthermore, the association of ATP-bound LinClpA^∆N^ with the LinClpP1P2 heterocomplex supported the degradation of eGFP-SsrA, albeit with 15% lower efficiency than the LinClpAP1P2 machinery after 60 minutes (Figure 8B).

**Table 1:** Comparison of the SsrA tag of *Leptospira* and other pathogenic bacteria

Organism	SsrA tag (number of residues)
*A. tumefaciens*	ANDNNAKEYALAA (13)
*B. subtilis*	GKTNSFNQNVALAA (14)
*C. difficile*	ADDNFAIAA (9)
*C. trachomatis*	AEPKAECEIISFADLEDLRVAA (22)
*E. coli*	ANDENYALAA (10)
*H. influenza*	ANDEQYALAA (10)
*H. pylori*	VNNTDYAPAYAKAA (14)
*L. interrogans*	ANNELALAA (9)
*L. monocytogenes*	GKEKQNLAFAA (11)
*M. tuberculosis*	ADSHQRLAA (9)
*N. meningitidis*	ANDETYALAA (10)
*P. aeruginosa*	ANDDNYALAA (10)
*S. aureus*	GKSNNNFAVAA (11)
*S. mutans*	AKNTNSYAVAA (11)
*S. pneumoniae*	AKNNTSYALAA (11)
*S. pyogenes*	AKNTNSYALAA (11)
*S. typhimurium*	ANDETYALAA (10)
*X. campestris*	ANDDNYGSDFAIAA (14)

In *E. coli*, the EcoClpS has been reported to redirect the specificity of the EcoClpAP protease complex away from SsrA-tagged substrates by significantly inhibiting their degradation [17]. Therefore, we conducted a comparison of the degradation of the eGFP-SsrA substrate by the LinClpAP1P2 machinery (3 µM) in the absence and presence of adaptor proteins (LinClpS1 and LinClpS2; 5 µM) (Figure 8C). As expected, the presence of LinClpS1 and LinClpS2 reduced the degradation of the eGFP-SsrA substrate to 53% and 39%, respectively, compared with LinClpAP1P2 machinery (100%). To achieve complete inhibition of LinClpAP1P2 activity, we introduced increasing concentrations of LinClpS1 and LinClpS2 (5, 10 and 15 µM) into the reaction mixture and subsequently monitored the degradation rate of eGFP-SsrA (Figure 8D). Significantly, the addition of 15 µM LinClpS1 and 10 µM LinClpS2 resulted in a total suppression of the proteolytic activity of the LinClpAP1P2 complex against the SsrA-tagged model substrate. To investigate how the LinClpA^∆N^ would influence this inhibitory effect of adaptor proteins, the degradation of eGFP-SsrA by LinClpA^∆N^P1P2 in the absence and presence of adaptor proteins was measured. In contrast with LinClpA, the LinClpA^∆N^, in association with the LinClpP1P2 heterocomplex, showed full activity on eGFP-SsrA measured after 60 minutes of degradation at 37°C in the presence of LinClpS1 and LinClpS2 (Figure 8C).

To further validate the functionality of the determined SsrA tag of *Leptospira*, another model protein substrate was generated by adding the 9 residues (ANNELALAA) SsrA tag at the C-terminal of an outer membrane leptospiral protein (LIC13341) previously characterized in our laboratory [68]. The recombinant LIC13341-SsrA substrate was purified, and its time-dependent degradation was analysed using ATP-bound LinClpAP1P2 machinery. Interestingly, the LIC13341-SsrA tagged substrate remained stable over 180 minutes when visualized on SDS-PAGE by Coomassie staining (Supplementary Figure S6C). When the model substrate (LIC13341-SsrA) was not recognized by the LinClpAP1P2 machinery, it led to the auto-degradation of LinClpA in a time-dependent manner (Supplementary Figure S6C). This study indicates that simply tagging any protein with the cognate SsrA tag is not enough for it to be recognized by the Clp chaperone–protease complex. Additional specificity factors within the substrates are likely necessary and should be thoroughly validated in future research.

## Discussion

The Clp family of proteins is widely present across different life forms and plays a crucial role in the general stress response and the virulence of many pathogenic bacteria [42]. In the genome of *L. interrogans*, several typical Clp family proteins have been predicted, including ClpS, ClpA, ClpX, ClpP, ClpQ and ClpY. The *Leptospira* species possess a diderm structure, including an outer membrane containing lipopolysaccharide (LPS), linking them to gram-negative bacteria [42]. While most gram-negative bacteria have the class I ATPase chaperone ClpA but lack ClpC chaperone, the *Leptospira* species contain both ClpA and ClpC ATPase chaperones [42]. The functionality of ClpC chaperone and its association with Clp protease have been well described in organisms such as *S. aureus*, *B. subtilis*, *M. tuberculosis* and *L. interrogans* [36,61,69,70]. However, the detailed biochemical characterization of ClpA in complex with Clp protease and adaptor proteins has only been limited to *E. coli* [11].

The assembly of the Clp ATPase chaperone into hexamers is essential for its functionality [49]. Multiple studies have investigated nucleotide-induced oligomerization of EcoClpA, yielding inconsistent findings. According to a study by Maurizi and colleagues, the hexameric assembly of EcoClpA is contingent solely upon the binding of adenosine triphosphate (ATP) [49]. Conversely, another study suggested that EcoClpA oligomerization is not exclusively dependent on the presence of the γ phosphate of ATP. The presence of ATPγS, AMP-PNP, AMP-PCP, ADP·BeF and ADP all facilitate the formation of a hexameric EcoClpA [52]. Similarly, the oligomerization of LinClpA was not solely restricted to the presence of nucleotide triphosphates. Adenosine diphosphate (ADP) also promotes LinClpA oligomerization, albeit to a lesser extent than ATP. Intriguingly, this study recorded a shift in the oligomerization propensity of LinClpA in the presence of polyphosphate (PolyP). In bacterial cells, the accumulation of inorganic polyP occurs during oxidative stress or amino acid starvation [71]. In certain bacteria, such as *C. crescentus* and *P. aeruginosa*, polyP regulates various cell cycle events during stress conditions [72]. In *E. coli*, it has been reported that polyP interacts with the Lon protease via its ATPase domain, thereby regulating its activity [71]. However, the effect of polyP on other bacterial cell proteases has not been explored. While this study provided indications of the binding of polyP with the ATPase chaperone LinClpA and its promotion of oligomerization, a more comprehensive analysis is required to grasp the significance of polyP in leptospiral cell survival under stress conditions.

Biochemical analysis of LinClpA displayed optimal ATPase activity at pH 7 and a temperature of 25°C in the presence of specific divalent metals (Mg^2+^, Mn^2+^ and Co^2+^), with the highest activity observed with Mg^2+^. In contrast, both EcoClpA and EcoClpB exhibit ATPase activity in the presence of Mg^2+^, Mn^2+^ and Ca^2+^, while SmuClpL and SpnClpL demonstrate ATPase activity with Mg^2+^ and Mn^2+^ only [73–76]. Furthermore, like EcoClpB, LinClpC and SpnClpL, LinClpA hydrolyzes various nucleotides (ATP, GTP, CTP and UTP) [36,74,76]. However, the phosphate hydrolysis activity of EcoClpA is limited to ATP only [73]. The *in silico* analysis of LinClpA indicates the conservation of crucial elements including the Walker A and Walker B motifs, Arginine finger, Sensor I and Sensor II motifs, underscoring their significance in Clp ATPase activity. A comparison of the amino acid sequence of the N-domain of LinClpA with EcoClpA reveals an approximate 40% sequence identity. Additionally, the tertiary structure analysis of the modelled LinClpA N-domain using AlphaFold demonstrates a high degree of structural similarity with the crystal structure of the EcoClpA N-domain.

In the context of *E. coli*, there exists substantial variation in studies characterizing different N-domain-deleted EcoClpA variants. The EcoClpA N-domain comprises 143 residues with an attached linker region of 24 residues, followed by two ATPase domains [19]. One study focusses on an EcoClpA variant (EcoClpA^∆168^) lacking 1–168 residues from the N-terminal, which exhibited a 20-fold reduction in ATPase activity. However, the complex formed with EcoClpP (EcoClpA^∆168^P) was inactive in degrading the casein model substrate [77]. Similarly, another variant of EcoClpA (EcoClpA^∆161^) recorded a 50% reduction in ATPase activity; nevertheless, the EcoClpA^∆161^P complex degraded casein and SsrA-tagged substrates at a 20-30% reduced rate [78]. Conversely, another EcoClpA^∆153^ variant exhibited higher ATPase activity and a higher rate of degradation of casein and GFP-SsrA substrates mediated by the EcoClpA^∆153^P complex [79]. Further, the N-terminal domain-deleted variant EcoClpA^∆143^ retained ATPase activity as EcoClpA but compromised the EcoClpA^∆143^P complex protease activity. The EcoClpA^∆143^P complex exhibited a slower rate of degradation of GFP-SsrA than EcoClpAP [80].

A recent study showed that the deletion of the N-domain and complete linker region (EcoClpA^∆N+∆L^) did not alter its ATPase activity but increased the catalytic efficiency of the complex EcoClpA^∆N+∆L^P in degrading the cognate GFP-SsrA substrate [53]. On the other hand, in another variant EcoClpA^∆L^, where only the linker region was deleted, the ATPase activity of chaperone and the degradation rate of GFP-SsrA mediated by the complex EcoClpA^∆L^P was retained [53].

In this study, a deletion variant of LinClpA has been generated where the predicted N-domain (1–138 residues) has been deleted, and thus, the LinClpA^∆N^ comprises a linker region (22 residues) and the 2 ATPase domains. Similar to earlier reported variants of EcoClpA (ClpA^∆N+∆L^ and ClpA^∆143^), the LinClpA^∆N^ efficiently assembled into higher-order structures in the presence of ATP [53,80]. This indicates that the N-domain has no role in ATP-induced oligomerization of ATPase proteins. Moreover, the LinClpA^∆N^ exhibits a higher ATPase activity, which agrees with the EcoClpA^∆153^ variant study [79]. However, other reported EcoClpA N-domain variants exhibit either similar (ClpA^∆N+∆L^ and ClpA^∆L^) or reduced (ClpA^∆168^ and ClpA^∆161^) ATPase activity to wildtype EcoClpA [53,77,78].

Next, the studies concerning the association of EcoClpP with N-domain variants of EcoClpA show discrepancies. The association between EcoClpA^∆143^ and EcoClpP is reported to be less stable than the EcoClpAP association as the addition of EcoClpP to EcoClpA^∆143^ failed to stimulate its ATPase activity [80]. However, another study shows that the EcoClpA^∆N+∆L^, EcoClpA^∆L^ and EcoClpA show a similar ability to associate with EcoClpP [53]. Notably, the LinClpA or its deletion variant (LinClpA^∆N^) shows a similar affinity for interaction towards LinClpP isoforms (ClpP1 and ClpP2) in this study. Thus, it can be ascertained that the deletion of the LinClpA N-domain does not influence the ability of Clp chaperone–protease complex formation.

The structural superimposition of modelled LinClpA with the crystal structure of EcoClpA (PDB: 1KSF) shows that the N-domain residues which are reported to be important for binding with the adaptor proteins in EcoClpA N-domain (Thr81, Glu23, Glu28 and Arg86) are conserved in LinClpA N-domain (Thr76, Glu18, Glu23 and His81) [22]. The adaptor proteins serve as substrate recognition components and deliver the recognized substrate to their cognate ATPase chaperone [81]. The EcoClpS adaptor exhibits dual functionality. EcoClpS acts as either an efficient stimulator for the degradation of N-degron substrates or as an inhibitor for the degradation of C-degron (SsrA-tagged) substrates by EcoClpAP [81]. The core region of EcoClpS binds to the N-domain of EcoClpA via a salt bridge and hydrogen bonds while the NTE region of EcoClpS engages with the EcoClpA ATPase domain. The EcoClpS NTE engagement with the EcoClpA translocation channel suppresses its ATPase activity [82]. Without EcoClpS, the EcoClpAP efficiently degrades SsrA-tagged substrates more than N-degron substrates. When EcoClpS is present, its core region has hydrophobic binding pockets for N-degrons, facilitating enhanced delivery of N-degron substrates to the EcoClpAP machinery. Also, EcoClpS decreases the affinity of SsrA tagged substrates to the EcoClpA by a non-competitive binding mechanism [82]. Thus, the EcoClpS binding to the EcoClpA switches the substrate specificity of complex EcoClpAP from SsrA-tagged substrates to N-degron substrates [82].

The Clp system of *Leptospira* possesses two adaptor proteins (LinClpS1 and LinClpS2), which share 17% sequence identity among themselves while having 23% and 42% sequence identity with EcoClpS, respectively. The LinClpS residues predicted to be important for the association with LinClpA N-domain are more conserved in LinClpS2 than in LinClpS1. These variations within the chaperone binding pocket of LinClpS1 and LinClpS2 might hint towards their different affinity for the LinClpA N-domain. The determined *Kd* for the LinClpAS2 complex (0.32 µM) was similar to the reported [22] *Kd* for the EcoClpAS complex (0.33 µM), while a higher *Kd* (0.44 µM) was observed for the LinClpAS1 complex. Further, a dose-dependent inhibition of ATPase activity in LinClpA was observed in the presence of LinClpS1 and LinClpS2. Although LinClpS1 forms a less stable complex with LinClpA, its presence (10 µM) inhibits 57% ATPase activity in LinClpA. The stable complex formation between LinClpA and LinClpS2 results in more than 90% ATPase activity inhibition in LinClpA. Moreover, no inhibitory effect of either LinClpS1 or LinClpS2 on the ATPase activity of variant LinClpA^∆N^ was observed, implying that LinClpS can only interact with LinClpA with intact N-domain.

Apart from mediating the degradation of SsrA-tagged or N-degron protein substrates, the EcoClpA serves as a substrate and undergoes proteolytic cleavage mediated by EcoClpAP. The EcoClpA was reported to undergo such auto-degradation to regulate the levels of EcoClpA protein *in vivo* [54]. The EcoClpA undergoes EcoClpAP-mediated auto-degradation *in vitro* with a half-life of 15 minutes, and the binding of EcoClpS prevents the auto-degradation process [17]. In line with earlier findings in *E. coli*, the LinClpA also undergoes auto-degradation by LinClpAP1P2 machinery under *in vitro* conditions with a half-life of approximately 60 minutes. As contemplated, in the presence of adaptor proteins (LinClpS1 and LinClpS2), the auto-degradation of LinClpA by protease complex is inhibited.

Additionally, the functionality of the predicted SsrA-tag sequence (ANNELALAA) in *Leptospira* was explored in this study. The LinClpAP1P2 machinery degraded the cognate eGFP-SsrA protein substrate under *in vitro* conditions. The model eGFP protein lacking LinSsrA tag remained stable for 60 minutes of reaction time under similar conditions with LinClpAP1P2 machinery and agrees with earlier reported studies on EcoClpAP and GFP [83]. Also, LinClpS binding to the N-domain of LinClpA inhibits SsrA-tagged protein degradation. In contrast, adding LinClpS to LinClpA^∆N^P1P2 machinery did not affect the degradation rate of cognate eGFP-SsrA. Thus, the N-domain of LinClpA is considered indispensable for adaptor protein-mediated regulation of LinClpAP1P2 activity. A recent study reports that GFP protein contains nine unstructured amino acids (THGMDELYK) at its C-terminal, and the insertion of *E. coli* SsrA tag (AANDENYALAA; 11 aa) extends the unstructured region to a total of 20 residues at the C-terminus and thus renders it as a model substrate for EcoClpAP [84]. However, a variant of GFP (lacking the nine unstructured C-terminal aa) tagged with EcoSsrA remained resistant to degradation by EcoClpAP. This resistance is likely due to the inability of the 11-residue long EcoSsrA tag to access the binding sites within the axial channel of EcoClpA [84]. In our study, the C-terminal LinSsrA tagging of the protein LIC13341 does not support its degradation by LinClpAP1P2 complex. The inability of the LIC13341-SsrA protein to be degraded by the LinClpAP1P2 complex might be due to limited accessibility of the 10-aa long LinSsrA tag to the key binding sites within the axial channel of LinClpA and it requires an additional unstructured region at the C-terminal end of protein substrates. In summary, this study reports the functional characterization of the molecular chaperone, LinClpA, and modulation of its activity upon binding with adaptor proteins (LinClpS1 and LinClpS2) and protease isoforms (LinClpP1 and LinClpP2).

## Materials and methods

### Bioinformatics analysis

The amino acid sequences of leptospiral ClpA, ClpS1 and ClpS2 proteins were compared using the protein Blast (Blastp) online server [85]. The sequences of ClpA (*L. interrogans* ClpA; Q72RD2, *E. coli* ClpA; P0ABH9, *X. campestris* ClpA; Q8P998, *B. burgdorferi* ClpA; O51342, *H. pylori* ClpA; A0AB33XJV6 and *P. aeruginosa* ClpA; Q9I0L8) and ClpS (*L. interrogans* ClpS1: Q72SM3, LinClpS2; Q72RD1, *E. coli* ClpS; P0A8Q6, *C. crescentus* ClpS; B8GZM8, *M. tuberculosis* ClpS; P9WPC0, *A. tumefaciens* ClpS1; Q8UFN4, AtuClpS2; Q8UD95, *S. elongatus* ClpS1; Q31QE7 and SelClpS2; Q31R11) from various pathogenic bacteria were obtained from the UniProtKB database [86]. A multiple sequence alignment was conducted using Clustal Omega software and analysed with the online tool ESPript (Easy Sequencing in PostScript) [87,88]. The tertiary structures of LinClpA, LinClpS1 and LinClpS2 were retrieved from the AlphaFold (AF) protein structure database [89]. The available structures of EcoClpA (1KSF) and EcoClpS (3O2O) were obtained from the protein data bank (PDB) [90]. Structural visualizations and superimpositions were performed using PyMol [91].

### Cloning, overexpression and purification of recombinant proteins

The full-length genes (*clpA; LIC11814*; 2220 bp, *clpS1; LIC11356*; 318 bp, *clpS2; LIC11815*; 333 bp) and partial open reading frame of *clpA* (*clpA^∆N^*; 1806 bp) lacking the sequence encoding N-domain of LinClpA of *L. interrogans* serovar Copenhageni strain Fiocruz L1-130 were amplified using PCR with its genomic DNA as a template. The oligonucleotides used for PCR amplification were designed based on the genomic sequence of *L. interrogan*s available on the National Center for Biotechnology Information (NCBI) and are listed in Table 2. Each gene was individually cloned into the pET-23a vector at the *Nhe*I and *Xho*I restriction sites to overexpress recombinant proteins with a C-terminal 6 × His tag. The transformed recombinant vectors were introduced into *E. coli* BL21 (DE3) strain for induction, overexpression and purification of the protein of interest. The transformed bacterial cells were cultured at 37°C in Luria-Bertani medium, and protein overexpression was induced by isopropyl β-D-1-thiogalactopyranoside (IPTG; 1 mM) in the pilot experiments to get the best yield during purification. For LinClpA and LinClpA^∆N^, the overexpression was induced for 16 hours at 18°C, while for LinClpS1 and LinClpS2, induction was performed for 4 hours at 37°C. The recombinant proteins were purified by nickel–nitrilotriacetic acid (Ni-NTA) affinity column chromatography under native conditions, as previously described [44]. The purified proteins were visualized on a 12% sodium dodecyl sulphate–polyacrylamide gel by Coomassie staining.

**Table 2:** Primers used in this study

Primer name	Primer sequence (5′-3′)
ClpA_F	CTAGCTAGCATGGAACGTACTTTAAGAAAGGCTT
ClpA_R	CCGCTCGAGATTCTTTTTTCCGGAAGAGAAT
ClpS1_F	CTAGCTAGCATGGCGAGTACACAAACTCCAG
ClpS1_R	CCGCTCGAGATCTTTCCAATGTAGCACTCA
ClpS2_F	CTAGCTAGCATGAGTGATATCTTTCGATTCGA
ClpS2_R	CCGCTCGAGTGACTCCTCCTCTCCTTCC
ClpA^∆N^_F	CGGCTAGCAAAGATTCTAAAAAAAATCCAGGC
ClpA^∆N^_R	CCGCTCGAGATTCTTTTTTCCGGAAGAGAAT
eGFP-SsrA_F	gccctggccgcctaaGAATTCGAGCTCCGTCGA
eGFP-SsrA_R	cagctcgttgttggcCTTGTACAGCTCGTCCATG
LIC13341-SsrA_F	gccctggccgcctaaTGACATCATCATCATCATCAC
LIC13341-SsrA_R	cagctcgttgttggcCTCGAGTTCTTGCTTGGAAAC

Nucleotide sequences in lowercase letters denote the inserted nucleotides.

### Generation of Anti-LinClpA antibodies and immunoblot analysis

Polyclonal antibodies specific to purified LinClpA of *L. interrogans* were generated in mice as described previously [36]. Briefly, BALB/c mice (4–6 weeks old, *n* = 5) were immunized with purified LinClpA (15–20 μg) emulsified in Freund’s complete adjuvant (FCA; Santa Cruz Biotechnology #SC-3727). The primary immunization was administered subcutaneously, followed by two booster injections of LinClpA emulsified in Freund’s incomplete adjuvant FIA; Santa Cruz Biotechnology #SC-3726 at days 14 and 21 of primary immunization. Blood was collected by retro-orbital bleeding seven days after the second booster and the sera were pooled. The LinClpA antibody titre was analysed by ELISA. The immunization experiments were performed at the Department of Microbiology, College of Veterinary Science, Assam Agricultural University, Guwahati, India, after approval by the Institutional Animal Ethics Committee (approval no.770/ac/CPCSEA/FVSC/AAU/IAEC/13–14).

To detect native LinClpA expression in *L. interrogans* serovar Copenhageni, whole-cell lysates of 3 × 10^9^ spirochetes were re-suspended in sodium dodecyl sulphate (SDS) loading dye. The pure recombinant LinClpA protein and lysate of *Leptospira* were resolved in 12% SDS-PAGE and transferred to a nitrocellulose membrane (Bio-Rad). The membranes were blocked with 5% non-fat dried milk prepared in Tris-buffered saline (TBS; pH 8.0) containing 0.1% Tween 20 (TBS-T) and probed with mouse anti-LinClpA (1:1000) antibodies for 2 hours at room temperature. After being washed, the membranes were incubated with horse radish peroxidase (HRP)-conjugated goat anti-mice IgG (1:5000; Sigma) for 1 hour, and immunoblots were developed by adding the chemiluminescence substrate (Thermo Scientific, catalog no. 32209). All dilutions of antibodies were prepared using 2% non-fat dried milk in 0.1% TBS-T.

### Leptospiral SsrA-tag sequence prediction and generation of model substrates

The amino acid sequences of the SsrA tag of *L. interrogans* and several pathogenic bacteria were retrieved from the transfer-messenger RNA database (tmRDB) [64]. The last nine amino-acid sequences of predicted SsrA tag from several bacteria were analysed by the WebLogo web server [65]. Site-directed insertion mutagenesis was performed to generate model protein substrates with LinSsrA tag at the C-terminal end. A Q5 site-directed mutagenesis kit (NEB, catalog no. E0554S) was used to insert the 30-nucleotide sequence (GCCAACAACGAGCTGGCCCTGGCCGCCTAA) encoding 9-amino acid long SsrA tag (ANNELALAA) of *Leptospira* at the downstream of *eGFP* and *LIC13341* genes in the pET-21d and pET-28a vectors, respectively. The recombinant proteins (eGFP-SsrA; 30 kDa and LIC13341-SsrA; 43 kDa) were purified as previously described [68].

### Size exclusion chromatography

The recombinant LinClpA (approximately 3 μM) was carefully loaded onto a HiLoad™ 16/600 Superdex™ 200 pg column (GE Healthcare #28–9893-35) following equilibration of the column in a buffer composed of 50 mM Tris-HCl (pH 8), 300 mM NaCl and 10% glycerol. LinClpA was incubated with ATP (2 mM) for 20 minutes at 4°C in an assay buffer that contained 50 mM Tris-Cl (pH 7.8), 50 mM KCl, 1 mM DTT and 8 mM MgCl2. The ATP-bound LinClpA was then loaded onto the equilibrated column and eluted at a flow rate of 1 ml/min at room temperature. For the determination of oligomeric size, molecular weight standards (Sigma, catalog no. MWGF-200) included β-amylase (200 kDa), alcohol dehydrogenase (158 kDa) and albumin (66 kDa).

### ANS binding assay

The nucleotide-induced oligomerization tendency of LinClpA was assessed using a hydrophobic fluorophore, ANS, as outlined in a previous study [36]. LinClpA (3 μM) was pre-incubated with or without different nucleotide analogues (ATP, GTP, CTP, UTP, ADP, PolyP, AMP; 2 mM) for 20 minutes at 4°C in an assay buffer (50 mM Tris-Cl; pH 7.8, 50 mM KCl, 1 mM DTT and 8 mM MgCl_2_). The ATP-bound or pure LinClpA was then mixed with ANS (10 μM) and incubated for 30 minutes at room temperature in the dark. A control reaction with ANS (10 μM) in assay buffer was also kept under similar conditions. Fluorescence spectra were recorded in a black, flat-bottom 96-well microplate (Invitrogen) using a spectro-fluorometer (iTECAN infinite M PLEX). All samples were excited at 350 nm, and emission spectra were recorded from 400 to 750 nm with 5 nm increments.

### ATPase activity analysis

The ATPase activity was measured by determining the rate of release of free phosphate (μM min^-1^) upon ATP hydrolysis using a malachite green phosphate assay kit (Sigma; MAK307), as previously described [36]. Each 40 μl ATPase assay reaction contained the enzymes (LinClpA or LinClpA^∆N^; 1 μM) and the required amount of ATP (1–8 mM) in assay buffer and was incubated for 1 hour at 37°C. To study the effect of the presence of Clp protease and adaptor proteins on ATPase activity, the reactions containing LinClpA or LinClpA^∆N^ were pre-incubated with increasing concentrations (1–10 μM) of LinClpP (ClpP1 and ClpP2) and LinClpS (ClpS1 and ClpS2) proteins for 10 minutes at 37°C, followed by ATP addition. The absorbance measurements at 620 nm were performed using a spectrofluorometer (iTECAN infinite M PLEX). All experiments were performed independently three times and in duplicate.

### Immunoassay for interaction analysis

An indirect ELISA was conducted to examine the interaction between chaperone–protease (LinClpAP complex) and chaperone–adaptor proteins (LinClpAS complex). Test proteins (LinClpP1/LinClpP2/LinClpS1/LinClpS2; 1 μM/well) and a control protein (bovine serum albumin; BSA; 1 μM/well) were coated on a 96-well microtiter plate overnight at 4°C. Following this, each well was blocked with a 3% BSA solution in phosphate-buffered saline (PBS) for 2 hours at 37°C and then overlaid with increasing concentrations of ATP-bound LinClpA or LinClpA^∆N^ (0–3 μM) for 2 hours at 37°C. After three washes with PBS containing 0.05% Tween 20 (PBS-T) buffer, mouse anti-LinClpA (1:1000) was added. The interaction was detected using HRP-conjugated anti-mouse secondary antibodies (1:5000) with TMB (Trimethylbenzidine, Thermo Scientific) as the substrate. The absorbance was measured at a wavelength of 450 nm after terminating the reaction using 1 M H_2_SO_4_ as per the manufacturer’s instructions. All experiments were independently performed three times and in duplicate.

### Auto-degradation assay of LinClpA and its variant

The LinClpA or its N-domain-deleted variant (LinClpA^ΔN^), at a specific concentration (1.5 μM), was pre-incubated with either pure LinClpP isoforms (3 μM) or the LinClpP1P2 heterocomplex in assay buffer for 10 minutes at 37°C before the addition of ATP (5 mM). When necessary, individual adaptor proteins (LinClpS1 and LinClpS2, 5 μM) were added to the reaction mixture. The reactions were carried out for 180 minutes at 37°C, with 25 μl of the reaction mixture aliquoted at 0, 60, 120 and 180 minute intervals. These aliquots were mixed with 5 × SDS sample buffer and heated for 10 minutes at 95°C to halt the degradation reaction. Following separation by SDS-PAGE and Coomassie staining, the amount of LinClpA and LinClpA^ΔN^ remaining was quantified using densitometry analysis with Image Lab software from Bio-Rad.

### Degradation of SsrA-tagged substrates by LinClpAP1P2 protease machinery

For the protease assay, equimolar amounts of LinClpP1 and LinClpP2 were allowed to incubate for 10 minutes at 37°C to form the LinClpP1P2 heterocomplex. Following this, LinClpA was added to the self-assembled LinClpP1P2 heterocomplex and allowed to incubate for 10 minutes at 37°C to create the LinClpAP1P2 machinery. Whenever necessary, the individual adaptor proteins (LinClpS1 and LinClpS2) were added at different concentrations (5, 10, 15 μM). The proteolytic activity of the LinClpAP1P2 machinery (3 μM) was tested on the eGFP-SsrA model substrate (2 μM) in an assay buffer containing ATP (5 mM). The degradation of eGFP-SsrA was monitored in black, flat-bottom 96-well microplates (Invitrogen) at 37°C for 60 minutes by measuring the loss of fluorescence at an excitation wavelength of 485 nm and emission wavelength of 525 nm. The change in relative fluorescence units (∆RFU) for eGFP-SsrA (RFU_Initial_ - RFU_Final_) was determined and plotted against time.

Similarly, the LinClpAP1P2 machinery was allowed to degrade the LIC13341-SsrA substrate (2 μM) in assay buffer over 180 minutes at 37°C. At 0, 60, 120 and 180 minute intervals, 25 μl of the reaction mixture was sampled, mixed with SDS sample buffer and then visualized by running the aliquots on a 12% SDS-PAGE gel, followed by Coomassie staining.

## Supplementary Material

Online supplementary material

## Data Availability

All data are contained within the manuscript

## Open Access

This article has been published open access under our Subscribe to Open programme, made possible through the support of our subscribing institutions, learn more here: https://portlandpress.com/pages/open_access_options_and_prices#conditional

## Abbreviations

NTE: N-terminal extension
PolyP: polyphosphates
SDS-PAGE: Sodium Dodecyl Sulfate – Polyacrylamide Gel Electrophoresis
SsrA: small stable RNA A
eGFP: Enhanced Green Fluorescent Protein
tmRNA: transfer messenger RNA
