# Supplementary material for: The ClpA chaperone and the two adaptor proteins modulate the fate of the model substrate tagged with a SsrA-degron of Leptospira
Source: Biochem J. 2025 Aug 26;482(17):1253–75. doi: 10.1042/BCJ20253143 (PMC12493166; doi:10.1042/BCJ20253143)
Supplement: Online supplementary material [file bcj-482-17-BCJ20253143-s001.pdf]

Supplementary Information

The ClpA chaperone and the two adaptor proteins modulate the fate of the model substrate tagged with a SsrA-degron of *Leptospira*

Surbhi Kumari and Manish Kumar*

Department of Biosciences and Bioengineering, Indian Institute of Technology Guwahati, Guwahati -781039, Assam, India

*Corresponding author: Manish Kumar, Email: mkumar1@iitg.ac.in, Phone: +91-361-258-2230, Fax: +91-361-258-2249

Keywords: *Leptospira*, Caseinolytic protease, Clp ATPase, Adaptor proteins, SsrA-tagged substrates

Legend to figures

Fig. S1. The sequence comparison of *Leptospira* ClpA with bacterial orthologs. Multiple sequence alignment of ClpA protein sequences from *Leptospira interrogans* (LinClpA; Q72RD2), *Escherichia coli* (EcoClpA; P0ABH9), *Xanthomonas campestris* (XcaClpA; Q8P998), *Borrelia burgdorferi* (BbuClpA; O51342), *Helicobacter pylori* (HpyClpA; A0AB33XJV6) and *Pseudomonas aeruginosa* PaeClpA; Q9I0L8*)*. The conserved and semi-conserved regions are highlighted in red and yellow color, respectively.

Fig. S2. Tertiary structural comparison of N-domain, ATPase domain I and II of LinClpA **(A)** The structural superimposition of repeat 1 and 2 of the modeled LinClpA N-domain. The helices α1- α4 (repeat 1; pink) and α5- α8 (repeat 2; green) represents a pseudo-symmetry with r.m.s.d of 1.2. **(B)** The structural superimposition of modeled LinClpA ATPase domain I (orange) and EcoClpA ATPase domain I (Cyan; PDB: 1KSF) with r.m.s.d of 1. **(C)** The structural superimposition of modeled ATPase domain II of LinClpA (blue) and EcoClpA ATPase domain II (Magenta; PDB: 1KSF) with r.m.s.d of 1.3.

Fig. S3. Sequence comparison of ClpS orthologs and molecular characterization LinClp ATPase and adaptor proteins. **(A)** Heat map showing comparison of amino-acids sequence identity of LinClpS1; Q72SM3 and LinClpS2; Q72RD1 with ClpS proteins from various microorganisms, including *Escherichia coli* (EcoClpS; P0A8Q6), *Caulobacter crescentus* (CcrClpS; B8GZM8), *Mycobacterium tuberculosis* (MtuClpS; P9WPC0), *Agrobacterium tumefaciens* (AtuClpS1; Q8UFN4, AtuClpS2; Q8UD95), *Synechococcus elongatus* (SelClpS1; Q31QE7, SelClpS2; Q31R11). **(B)** The disorderdness of amino-acid sequences of LinClpS1 (106 aa), LinClpS2 (111 aa) and EcoClpS (106 aa) was determined using PrDOS software and the disordered probability was plotted with the respective residue number. The cut-off for the disordered region (0.5) is indicated with a dotted line. **(C)** Purification of LinClpA, LinClpA$^{\Delta N}$, LinClpS1 and LinClpS2 via Ni-NTA affinity chromatography. The purified recombinant

LinClpA (82 kDa), LinClpA$^{\Delta N}$ (66 kDa), LinClpS1 (12 kDa) and LinClpS2 (13 kDa) proteins resolved on 12 % SDS-PAGE stained with Coomassie blue. **(D)** Immunoblot analysis of *L. interrogans* serovar Copenhageni lysate. Lanes 1, 2 and 3 represent ladder, recombinant LinClpA and leptospiral whole cell lysate, respectively. The expression of native ClpA was detected in *Leptospira* lysate (lane 3) using mouse anti-LinClpA (1:1000 dilution). The recombinant LinClpA and native ClpA of *Leptospira* were detected at a similar molecular size (~ 82 kDa).

Fig. S4. Comparison of biochemical activity of LinClpA and LinClpA$^{\Delta N}$. (A) ANS binding assay to study the oligomerization of LinClpA in the absence (-) or presence (+) of various nucleotides (ATP, GTP, CTP and UTP). **(B)** Oligomerization of LinClpA and LinClpA$^{\Delta N}$ in the absence (-) or presence (+) of ATP. The fluorescence intensity of ANS was measured at an excitation wavelength of 350 nm and emission spectra in the range of 400-750 nm. **(C)** Effect of ATP at increasing concentration on the LinClpA and LinClpA$^{\Delta N}$ ATPase activity. The kinetics of the LinClpA and LinClpA$^{\Delta N}$ enzymatic reaction on ATP was determined by non-linear curve fitting in the Origin software, and the V_{max} and K_M values of the reaction were estimated.

Fig. S5. Immunoassay study using anti-LinClpA antibodies. (A) Direct ELISA was performed to check the specificity of the anti-LinClpA polyclonal antibody on the detection of various LinClp proteins (ClpP1/ClpP2/ClpS1/ClpS2/ClpA/ClpA$^{\Delta N}$). **(B)** Immunoassay for the interaction of LinClpA with LinClpP isoforms under in-vitro conditions in the presence and absence of various nucleotides (ATP, GTP, CTP and UTP).

Fig. S6. The comparison of SsrA-tag from various pathogenic bacteria and validation of leptospiral SsrA-tag. (A) WebLogo representation of SsrA-tag sequences from *Leptospira* and other pathogenic bacteria with a well-studied ClpP system. The sequences of SsrA-tag for various bacteria were retrieved from the tmRNA database, and the last 9 aa sequences were

selected for WebLogo representation. The degradation of the **(B)** eGFP-SsrA model substrate **(C)** LIC13341-SsrA substrate by the LinClpAP1P2 machinery. The time-dependent degradation of LinSsrA-tagged substrates was examined on 12 % SDS-PAGE within a time interval of 0, 60, 120 and 180 minutes.

Fig. S1

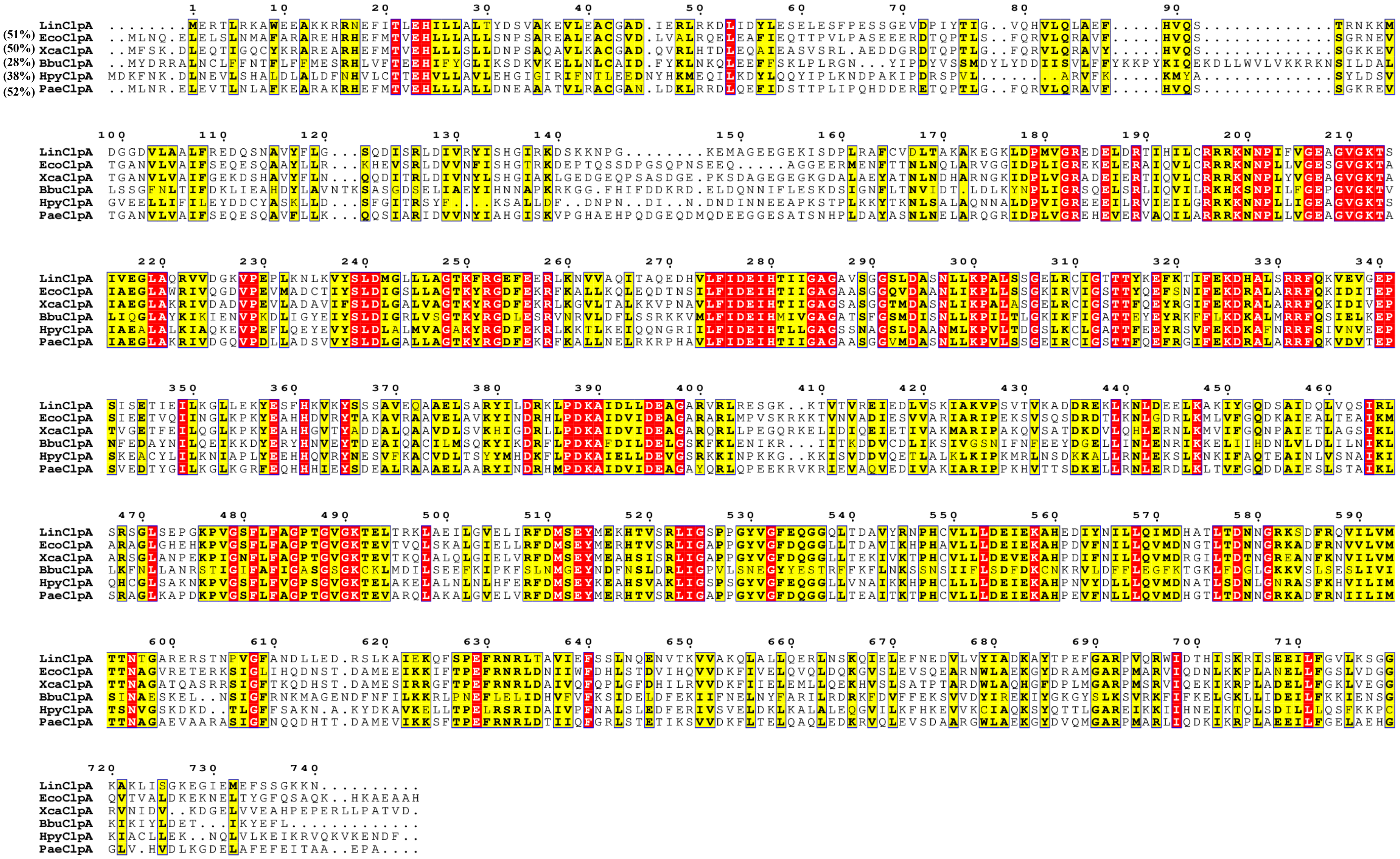

(51%) LinClpA
(50%) EcoClpA
(28%) XcaClpA
(38%) BbuClpA
(52%) HpyClpA
PaeClpA

Fig. S2

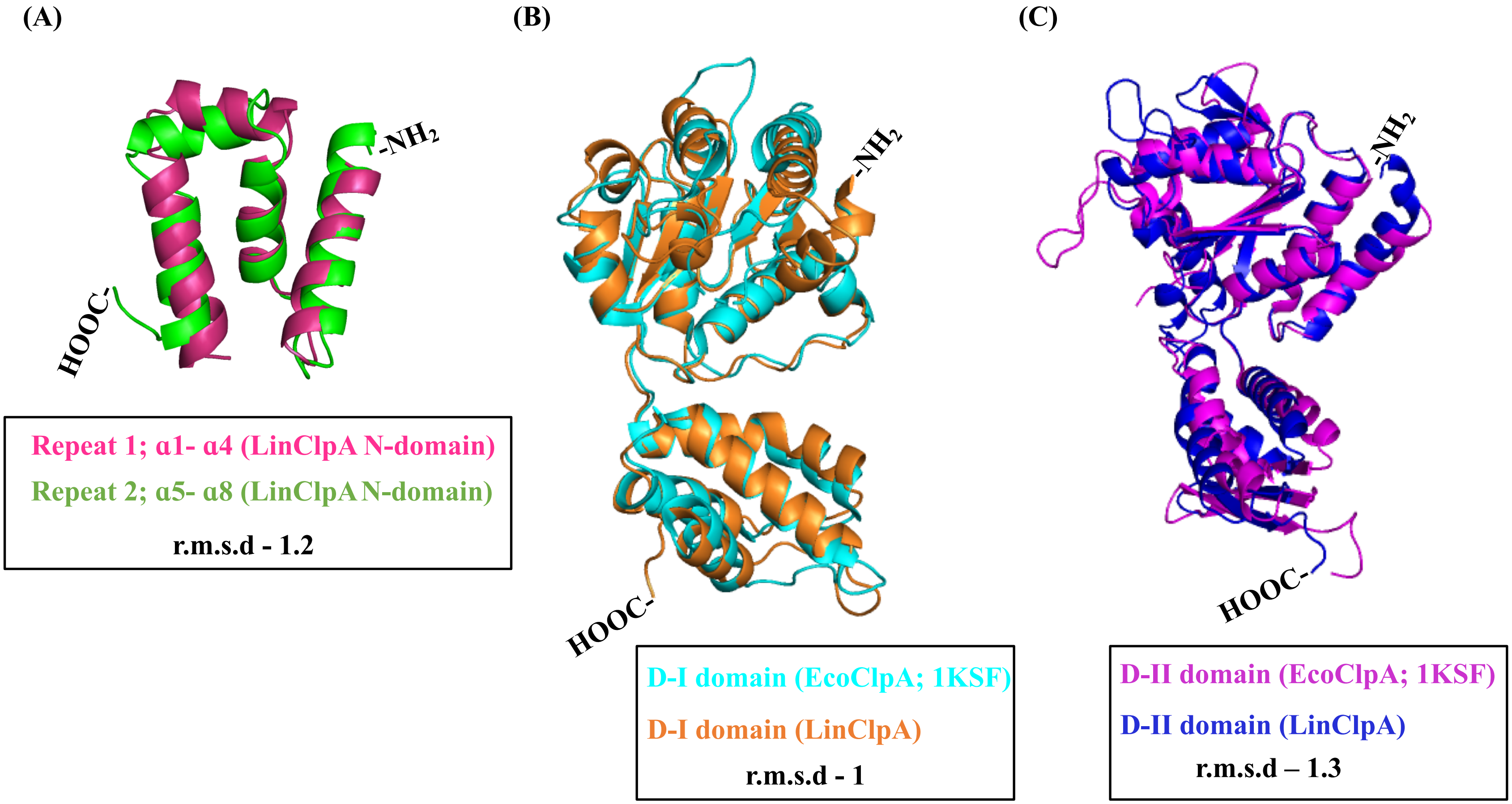
(A)
-NH2
HOOC-
Repeat 1; α1- α4 (LinClpA N-domain)
Repeat 2; α5- α8 (LinClpA N-domain)
r.m.s.d - 1.2
(B)
-NH2
HOOC-
D-I domain (EcoClpA; 1KSF)
D-I domain (LinClpA)
r.m.s.d - 1
(C)
-NH2
HOOC-
D-II domain (EcoClpA; 1KSF)
D-II domain (LinClpA)
r.m.s.d – 1.3

Fig. S3

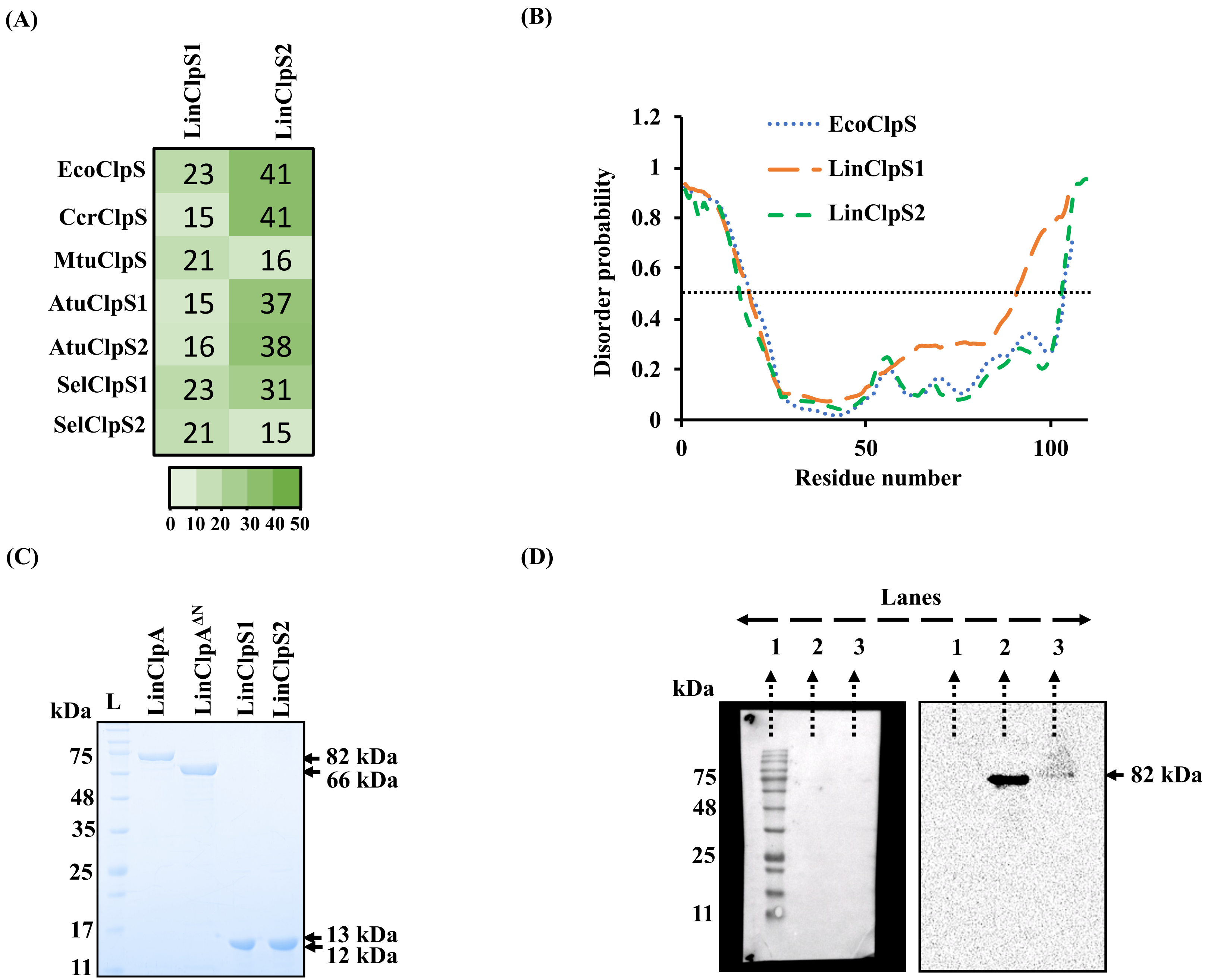
(A)
LinClpS1
LinClpS2
EcoClpS 23 41
CcrClpS 15 41
MtuClpS 21 16
AtuClpS1 15 37
AtuClpS2 16 38
SelClpS1 23 31
SelClpS2 21 15
0 10 20 30 40 50
(B)
EcoClpS
LinClpS1
LinClpS2
Disorder probability
1.2
1
0.8
0.6
0.4
0.2
0
0
50
100
Residue number
(C)
kDa
L
LinClpA
LinClpA^ΔN
LinClpS1
LinClpS2
75
48
35
25
17
11
82 kDa
66 kDa
13 kDa
12 kDa
(D)
Lanes
1 2 3 1 2 3
kDa
75
48
25
11
82 kDa

Fig. S4

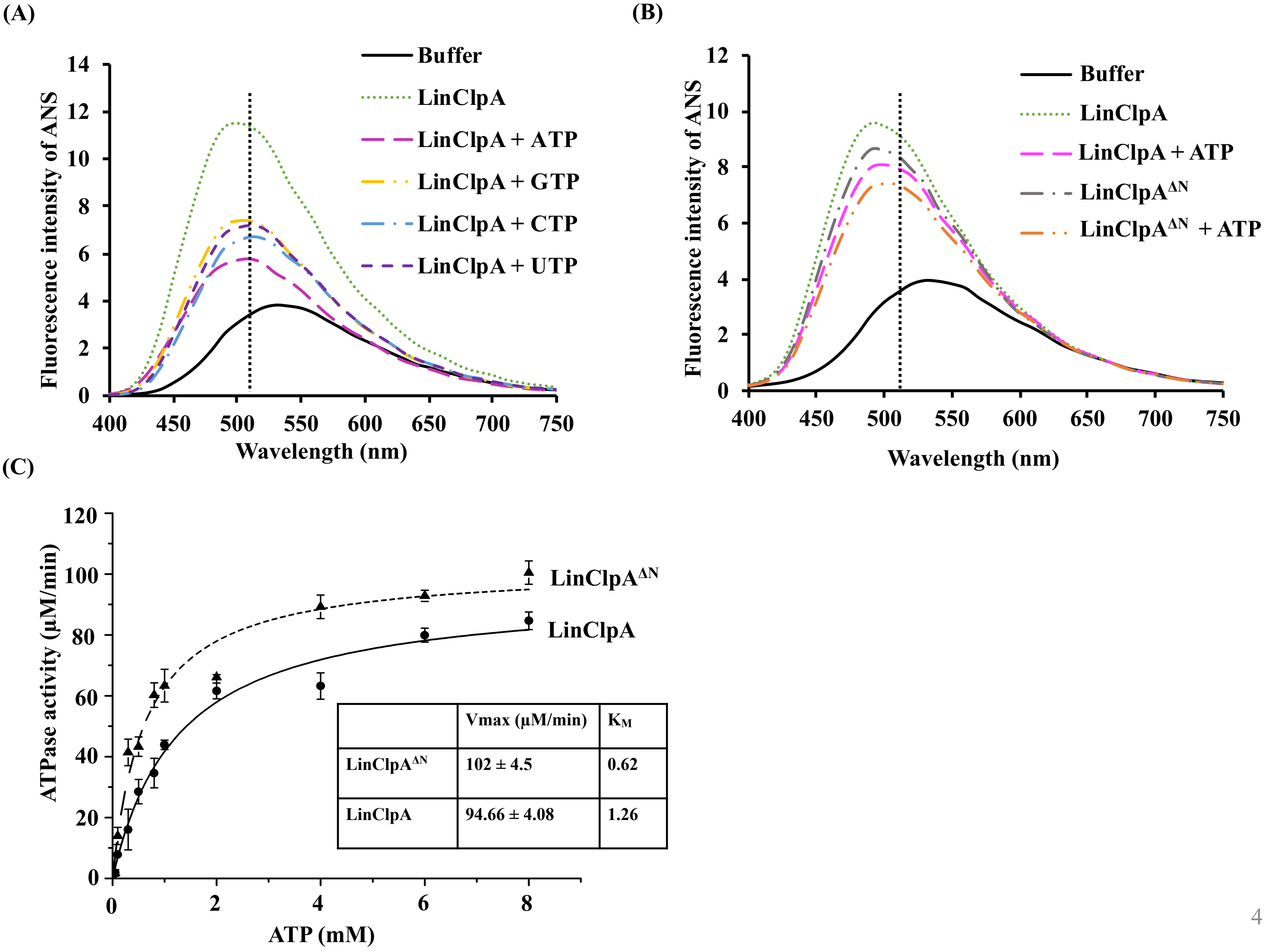

	Vmax (µM/min)	K_M
LinClpA$^{\Delta N}$	102 ± 4.5	0.62
LinClpA	94.66 ± 4.08	1.26

Fig. S5

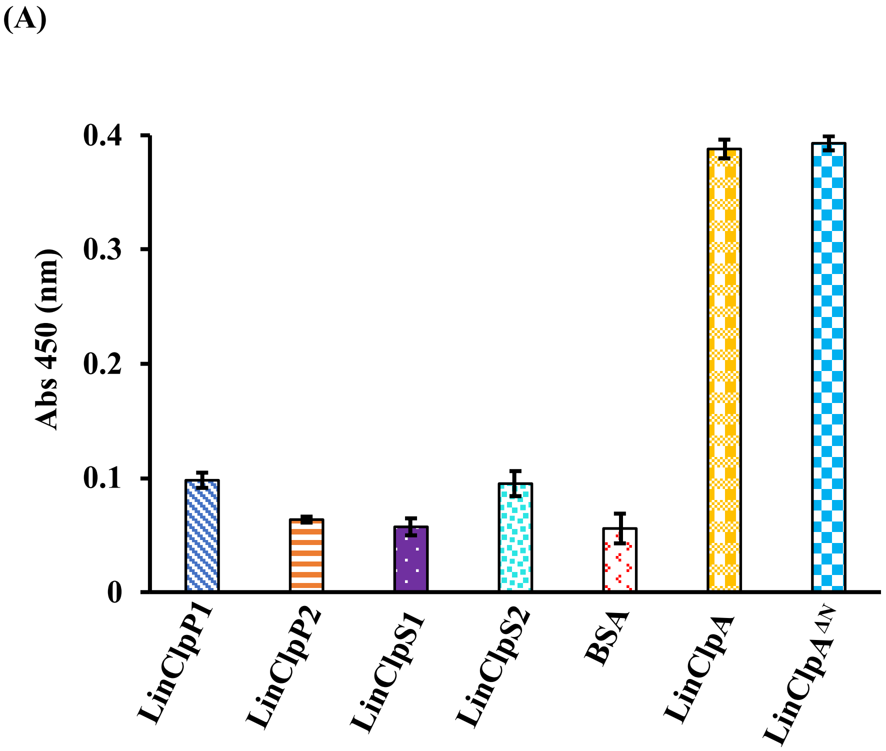
(A)
0.4
0.3
0.2
0.1
0
Abs 450 (nm)
LinClpP1
LinClpP2
LinClpS1
LinClpS2
BSA
LinClpA
LinClpA$^{\Delta N}$

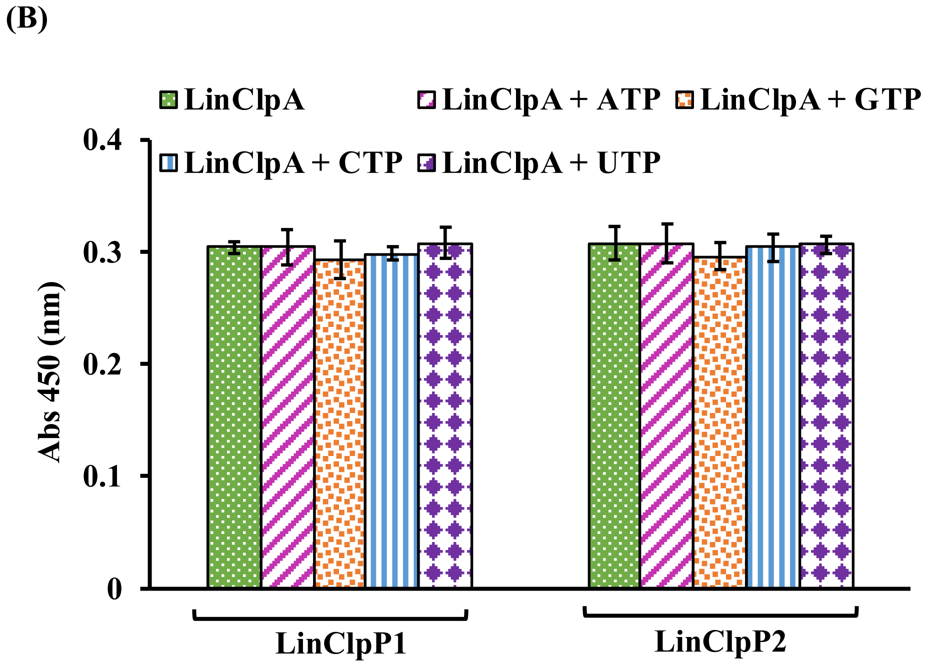
(B)
LinClpA
LinClpA + ATP
LinClpA + GTP
LinClpA + CTP
LinClpA + UTP
0.4
0.3
0.2
0.1
0
Abs 450 (nm)
LinClpP1
LinClpP2

Fig. S6

(A)

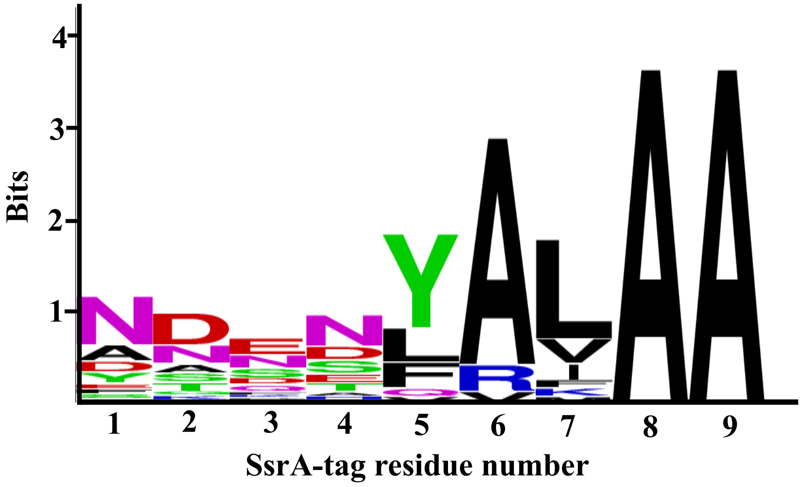
Bits
4
3
2
1
1
2
3
4
5
6
7
8
9
SsrA-tag residue number

(B)

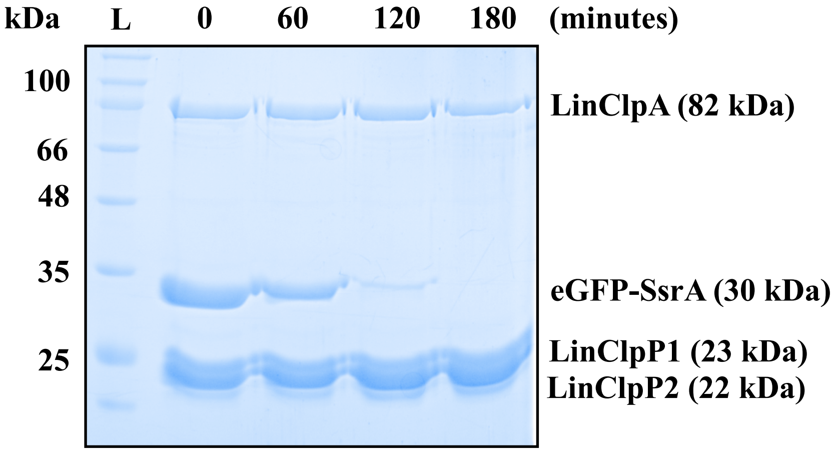
kDa
L
0
60
120
180
(minutes)
100
66
48
35
25
LinClpA (82 kDa)
eGFP-SsrA (30 kDa)
LinClpP1 (23 kDa)
LinClpP2 (22 kDa)

(C)

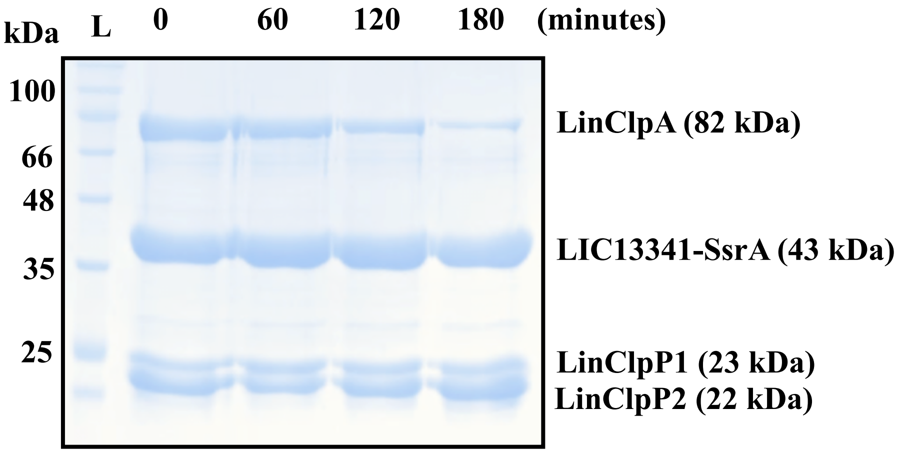
kDa
L
0
60
120
180
(minutes)
100
66
48
35
25
LinClpA (82 kDa)
LIC13341-SsrA (43 kDa)
LinClpP1 (23 kDa)
LinClpP2 (22 kDa)
